# A new cell culture resource for investigations of reptilian gene function

**DOI:** 10.1242/dev.204275

**Published:** 2024-11-22

**Authors:** Sukhada P. Samudra, Sungdae Park, Elizabeth A. Esser, Tryggvi P. McDonald, Arianna M. Borges, Jonathan Eggenschwiler, Douglas B. Menke

**Affiliations:** Department of Genetics, University of Georgia, Athens, GA 30602, USA

**Keywords:** Lizard, Anolis, Cell line, Hedgehog, CRISPR

## Abstract

The establishment of CRISPR/Cas9 gene editing in *Anolis sagrei* has positioned this species as a powerful model for studies of reptilian gene function. To enhance this model, we developed an immortalized lizard fibroblast cell line (ASEC-1) for the exploration of reptilian gene function in cellular processes. We demonstrate the use of this cell line by scrutinizing the role of primary cilia in lizard Hedgehog (Hh) signaling. Using CRISPR/Cas9 mutagenesis, we disrupted the *ift88* gene, which is required for ciliogenesis in diverse organisms. We determined that loss of *itf88* from lizard cells leads to an absence of primary cilia, a partial derepression of *gli1* transcription, and an inability of the cells to respond to the Smoothened agonist, SAG. Through a cross-species analysis of SAG-induced transcriptional responses in cultured limb bud cells, we further determined that ∼46% of genes induced as a response to Hh pathway activation in *A. sagrei* are also SAG responsive in *Mus musculus* limb bud cells. Our results highlight conserved and diverged aspects of Hh signaling in anoles and establish a new resource for investigations of reptilian gene function.

## Teaser

**Summary:** A new lizard embryonic fibroblast cell line is generated to enable *in vitro* studies of reptilian gene function.

## INTRODUCTION

The ability to manipulate genomes through transgenesis and targeted gene editing has been essential for advancing our understanding of gene function during development. Although these technologies have long been available in major model systems, the past decade has witnessed a rapid expansion in the number of species for which genome manipulation methods are available. One major group of amniotes in which gene function remains largely unexplored are squamate reptiles, the group that comprises lizards and snakes. Squamates are only distantly related to mammalian and avian models, having diverged from mammals approximately 320 million years ago and from avians 290 million years ago (Hedges et al., 2006). Furthermore, with over 11,000 described species (http://www.reptile-database.org), there is a tremendous amount of biology to explore within squamate reptiles. This provides strong motivation for the establishment of gene-editing methods and resources for studies of gene function in this clade.

The brown anole lizard, *Anolis sagrei*, was the first squamate species in which *in vivo* gene editing was reported, finally permitting the production of gene knockouts in a squamate model (Rasys et al., 2019). The recent publication of a high-quality reference genome for *A. sagrei* has further enhanced this model system (Geneva et al., 2022), as has the development of *ex ovo* whole embryo and *Anolis* limb micromass culturing methods (Infante et al., 2018; Park et al., 2014; Sanger and Kircher, 2017). Despite these advances, additional resources and tools are required for mechanistic studies of signaling pathways and cellular processes in *A. sagrei*. Here, we report the establishment and characterization of a new immortalized *A. sagrei* embryonic fibroblast cell line to enable experimental manipulation of gene function in cell culture. As proof of principle, we use this cell line to test the role of primary cilia in reptilian Hedgehog (Hh) signaling by using CRISPR/Cas9 to disrupt the *ift88* gene, which is required for the generation of primary cilia in diverse organisms (Han et al., 2003; Huang and Schier, 2009; Huangfu et al., 2003; Pazour et al., 2000, 2002; Qin et al., 2001). To complement these mechanistic studies of Hh signaling, we describe a simple procedure to induce Hh signaling *in ovo* using the Smoothened agonist SAG. In addition, we characterize the transcriptional responses of *Anolis* and mouse limb bud cells to SAG in cell culture to identify shared and species-specific Hh-responsive genes. Together, this work provides significant cell culture and transcriptome resources to advance studies of Hh signaling and other cellular processes in *Anolis* lizards.

## RESULTS

### Generation and characterization of an *A. sagrei* immortalized cell line

**Fig. 1.**
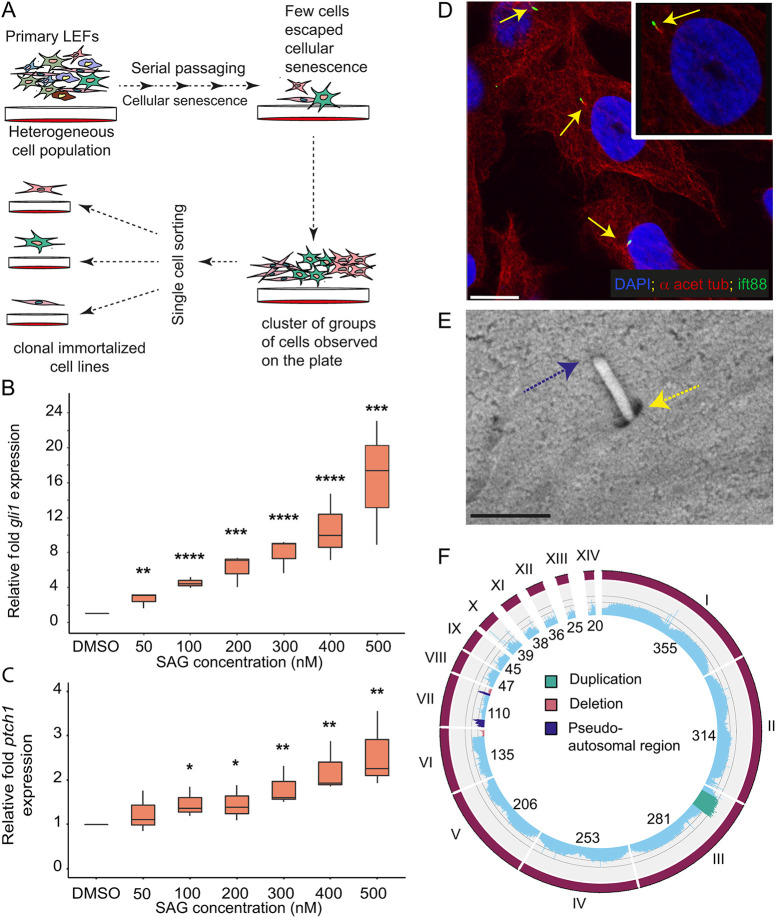
**Generation and characterization of the ASEC-1 lizard cell line.** (A) Process for the generation of immortalized cell lines from *Anolis* torso primary LEFs. (B,C) qRT-PCR showing relative *gli1* (B) or *ptch1* (C) expression in ASEC-1 cells in response to various SAG concentrations (*n*=3). Gene expression is relative to DMSO treated cells and is normalized against *tbp* and *atp5f1d*. *P<0.05, **P<0.01, ***P<0.001, ****P<0.0001 (paired *t*-test). The box limits represent the lower and upper quartile values of the dataset. The horizontal line represents the median value of the dataset. Whiskers mark the minimum and maximum values of the data points in the given dataset. (D) Visualization of the primary cilium on ASEC-1 cells by immunostaining for acetylated α-tubulin and Ift88. The primary cilium, indicated by yellow arrows, features a ciliary axoneme stained with acetylated α-tubulin antibody (red) and the tip of the cilium stained with Ift88 antibody (green). Scale bar: 10 µm. (E) SEM image of a primary cilium at 50,000× magnification. A yellow arrow points to the ciliary pit, and a blue arrow indicates the tip of the cilium. Scale bar: 1 µm. (F) Circular genome plot showing average WGS coverage over 1 MB bins for the ASEC-1 cell line and highlighting potential deletion and duplication events. Gray lines running parallel to axes represent 0.5×, 1×, and 1.5× of the genome wide WGS average. Scaffolds are labeled with Roman numerals.

To establish an immortalized *A. sagrei* cell line for functional genetic studies of Hh signaling and other biological processes in reptile cells, we employed a simple strategy whereby we serially passaged primary lizard embryonic fibroblasts (LEFs) collected from the torso region of a single stage 6 *A. sagrei* embryo (Fig. 1A). Cells entered senescence at passage 26, after which time a small fraction of cells resumed proliferation. Of these, 192 single cells were sorted into 96-well plates. This ultimately resulted in the generation of approximately 40 surviving cell lines from the *Anolis* embryo. To determine the suitability of these cells for studies of Hh signaling, we tested the responsiveness of three cell lines to SAG, which was validated as a potent inducer of the Hh pathway *in vivo* (see below). We exposed the cell lines to 200 nM SAG for 24 h; these conditions are sufficient to induce Hh signaling in immortalized mouse fibroblasts (Chen et al., 2002). In response to SAG exposure, all three clones demonstrated a significant increase in the expression of *gli1* and *ptch1*, known conserved transcriptional targets of the Hh pathway (Fig. S1A,B). We chose one of these SAG-responsive cell lines, *Anolis sagrei* embryonic cell line 1 (ASEC-1), to evaluate in greater detail. We observed that *gli1* and *ptch1* displayed a clear trend of elevated expression as SAG concentrations were increased (Fig. 1B,C). When exposed to 200 nM SAG for different time intervals from 6 to 72 h, the relative *gli1* expression intensified as exposure time increased (Fig. S1C,D). In both SAG dosage and time curve experiments, relative *gli1* expression was more sensitive to experimental conditions than was *ptch1* expression.

ASEC-1 was characterized with respect to cellular morphology, doubling time, density at confluence, survival after freezing, sensitivity to puromycin, and efficiency of transfection using cationic lipids (Fig. S2). Since Hh signaling is functionally linked to the primary cilium in vertebrates (Huangfu et al., 2003; Huangfu and Anderson, 2005), we used immunostaining and scanning electron microscopy (SEM) to examine ASEC-1 cells for the presence of primary cilia. We began by co-staining cells with antibodies raised against two established ciliary markers, acetylated α-tubulin and Ift88. The staining pattern was consistent with the presence of primary cilia (Robert et al., 2007; Snouffer et al., 2017), with each cell displaying, at most, a single co-stained structure with Ift88 staining occurring at the distal tip (Fig. 1D). To confirm the presence of primary cilia, we performed SEM on ASEC-1 cells (Fig. 1E). SEM images revealed the presence of a ∼1 µm long/∼0.3 µm diameter protrusion from the cells with a pit at the base of the protrusion, typical of fibroblast primary cilia.

To characterize the ASEC-1 cell line further, we assessed the transcriptome. RNA-sequencing (RNA-seq) analysis detected the presence of transcripts for common fibroblast markers, including *vim*, *col1a1*, *col1a2*, *col5a1*, *lum*, and the cell surface receptor *pdgfra* (Guerrero-Juarez et al., 2019; Meng et al., 2020; Muhl et al., 2020; Sunami et al., 2020)*.* Transcripts for common mural cell markers were also found, namely *des*, *mcam, notch3*, *pdgfrb*, and *anpep* (*cd13*) (Muhl et al., 2020; Sunami et al., 2020). The flattened, spindle-like morphology of the cells (Fig. S2A-C) combined with the presence of commonly expressed fibroblast molecular markers provides evidence for the fibroblast nature of the cell line (Table 1). RNA-seq analyses also confirmed that exposure of the cell line to SAG results in upregulation of *gli1* and *ptch1* (Table S1).

**Table 1.**
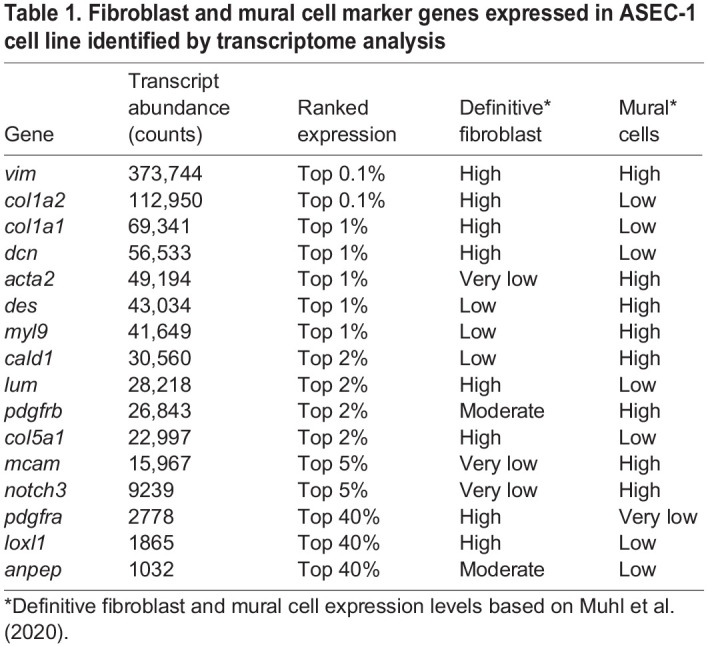
Fibroblast and mural cell marker genes expressed in ASEC-1 cell line identified by transcriptome analysis

To annotate sequence polymorphisms and detect major chromosomal abnormalities in the ASEC-1 cell line, we performed whole-genome shotgun sequencing and aligned the reads to a published *A. sagrei* reference assembly (Fig. 1F; Geneva et al., 2022). Sequence alignments revealed a large number of polymorphisms that distinguish the ASEC-1 cell line from the *A. sagrei* reference genome [16,101,990 single nucleotide polymorphisms (SNPs) and 862,358 indels]. The annotation of these polymorphic sites provides an important resource for the design of gene-editing experiments in ASEC-1. The published reference assembly (AnoSag2.1) was generated from a female and does not contain the *A. sagrei* Y chromosome. However, we found that the genome coverage of the X-specific portion of the X chromosome (i.e. the region of the X that has diverged from the Y chromosome) is half that of the autosomes, suggesting that the cell line is XY and derived from a male embryo. The presence of a Y chromosome was confirmed using PCR-based genotyping (Fig. S1E). Deviations from the average sequence coverage within a given chromosome indicate likely duplication or deletion events. We noted that scaffold 3 contains an apparent duplication of ∼42 Mb. This region displays 1.5 times the average genome-wide coverage. In addition, scaffold 6 exhibits an apparent monoallelic deletion of ∼20 Mb at one end of the chromosome. Scaffold 7, the X chromosome, also contains a putative monoallelic deletion of ∼14 Mb in a pseudoautosomal region. We found that genes encoding Hh ligands (*shh*, *ihh*, *dhh*), Gli proteins (*gli1*, *gli2*, *gli3*), Hh receptors (*ptch1*, *ptch2*), and *ift88*, a gene required for ciliogenesis, were not located in duplicated or deleted regions.

### Targeted disruption of *ift88* results in loss of the primary cilium

**Fig. 2.**
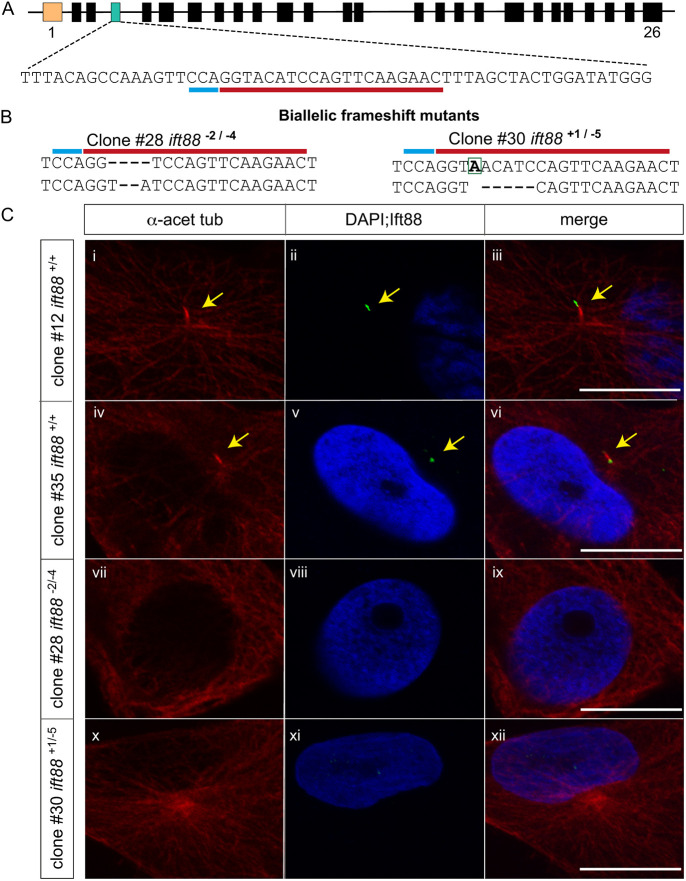
**CRISPR/Cas9 editing of the *ift88* gene in ASEC-1 cells.** (A) Schematic of the *A. sagrei ift88* gene (not to scale). Rectangles indicate exons. The first exon is noncoding and is indicated in orange. The fourth exon (green) was selected for gene editing. The targeted sequence is underlined in red with the protospacer adjacent motif (PAM) site underlined in blue. (B) Sequence analysis revealed biallelic indel mutations in clones #28 and #30. (C) Representative immunofluorescence images for detection of the primary cilium in wild-type and *ift88* knockout clones. In wild-type clones, acetylated α-tubulin antibody (red) stained the primary cilia axoneme with the Ift88 antibody (green) staining the tip of the cilium (i-vi). No obvious primary ciliary structures were observed in *ift88* knockout clones (vii-xii). Yellow arrows indicate primary cilia. Scale bars: 10 µm.

The *ift88* gene is required for ciliary assembly and maintenance in diverse organisms, ranging from *Chlamydomonas* to mouse (Huangfu and Anderson, 2005; Pazour et al., 2000, 2002). Therefore, to demonstrate the use of ASEC-1 for exploring gene function and to test the requirement of the primary cilium for Hh signaling in anoles, we disrupted the *ift88* gene in the ASEC-1 cell line by CRISPR/Cas9 gene editing. RNA-seq supported gene annotations (Geneva et al., 2022) indicate that the *A. sagrei ift88* gene contains 26 exons with the start codon located in exon 2 (Fig. 2A). We targeted exon 4 to disrupt the *ift88* open reading frame near the N terminus. After transfecting ASEC-1 cells with *Cas9* and *ift88* gRNA expression constructs, we sorted 192 individual cells into 96-well plates, which resulted in the successful isolation and propagation of 46 clonal cell lines. PCR amplification of exon 4 followed by PAGE revealed 19 clonal cell lines with indels in *ift88.* Sanger sequencing demonstrated that 12 of these clones carried frame-shifting mutations on both *ift88* alleles (Table S2). Additional sequencing of PCR-amplified *ift88* cDNA from mutant clones #28 and #30 confirmed the presence of biallelic frame-shifting mutations in *ift88* transcripts in these clones (Fig. 2B). In contrast, the chromatograms of two randomly selected clones (#12 and #35) that appeared wild type by PAGE analysis displayed no mutations.

The loss of *ift88* function in ASEC-1 cells is predicted to prevent ciliogenesis. By staining for acetylated α-tubulin, we could visualize the primary cilium in at least 70% of cells from the parental ASEC-1 cell line (Fig. 1D). Similarly, we could readily identify the presence of primary cilia in two control *ift88^+/+^* ASEC-1 clones (#12 and #35), which did not carry mutations in *ift88* (Fig. 2C). Co-staining with Ift88 antibody demonstrated that Ift88 is localized at the tip of the cilia in these clones. In contrast, cells from mutant clones (clone #28: *ift88^−2/−4^*; clone #30: *ift88^+1/−5^*) did not show any Ift88 staining. Moreover, based on acetylated α-tubulin, roughly 90% of the cells in clones #28 and #30 failed to show ciliary structures (Fig. 2C, vii-xii). The remaining cells showed ambiguous structures that could not be confidently categorized as primary cilia.

### *ift88* is required for SAG transcriptional responses

**Fig. 3.**
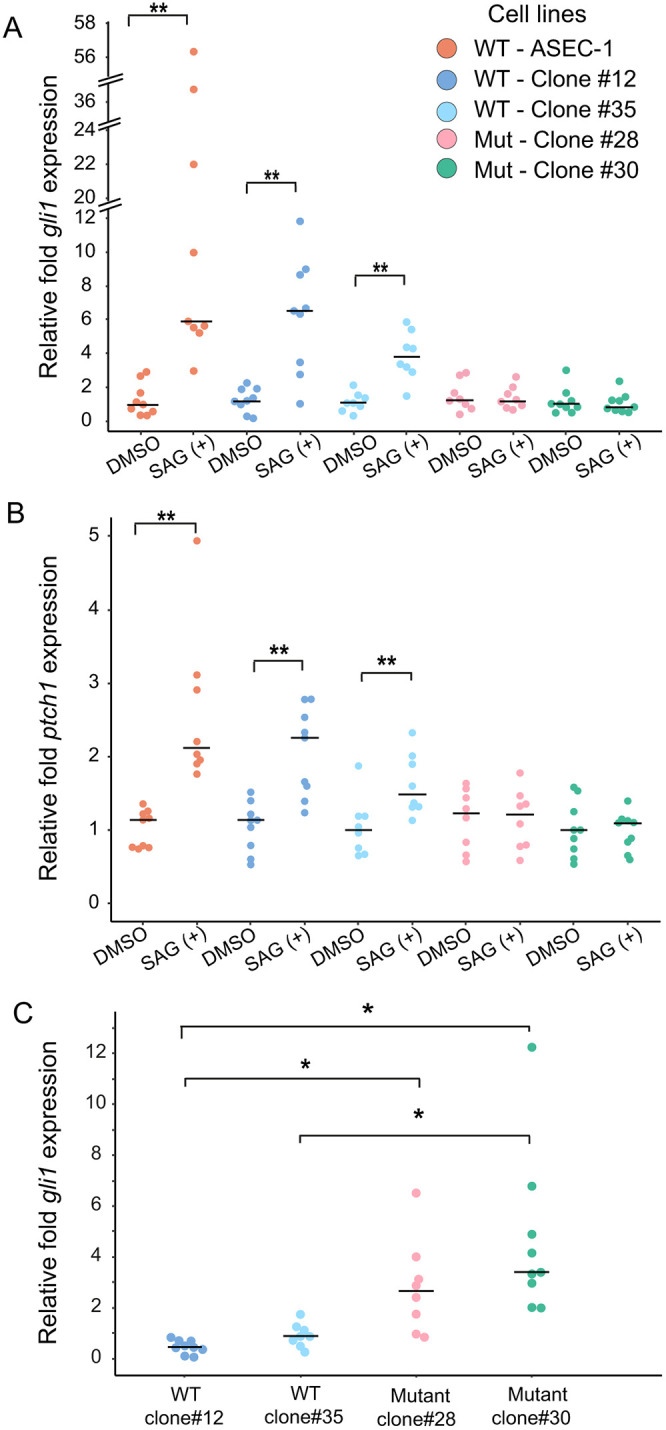
**Expression of *gli1* and *ptch1* in wild-type and *ift88* knockout ASEC-1 cells.** (A-C) Transcript levels were assessed by qRT-PCR. In each cell line, the induction of *gli1* (A) or *ptch1* (B) following SAG treatment was assessed relative to DMSO controls. The parental ASEC-1 cell line as well as two wild-type clones show an increase in *gli1* and *ptch1* expression in response to treatment with 400 nM SAG for 24 h. Mutant clones #28 and #30 showed no change in *gli1* or *ptch1* expression. (C) Relative basal *gli1* expression in DMSO-treated clones. Basal *gli1* expression is relative to the average ΔCt value of *gli1* expression in ASEC-1 DMSO-treated cells. *gli1* and *ptch1* expression was normalized to the two reference genes *tbp* and *atp5f1d*. *n*=9 for each condition. *P<0.05, **P<0.01 (A,B: Wilcoxon signed-rank test; C: Kruskal–Wallis rank sum test and Kruskal-Wallis multiple comparison test). Horizontal lines represent median values. Each individual data point represents a biological replicate.

To determine whether the loss of *ift88* and primary cilia affects Hh signaling, we studied SAG responsiveness of *ift88* mutant cell lines. The SAG responsiveness of wild-type ASEC-1 cells, as assessed by *gli1* and *ptch1* induction, was robust at 400 nM SAG concentration and 24 h of SAG exposure (Fig. 1B,C; Fig. S1). Hence, we chose to compare the SAG response of the parental cell line ASEC-1, clone #12 *ift88^+/+^*, clone #35 *ift88^+/+^*, clone #28 *ift88^−2/−4^*, and clone #30 *ift88^+1/−^*^5^ exposed to 400 nM SAG for 24 h. For each cell line, vehicle (DMSO) alone was used as a control treatment. Both of the wild-type clones had an increase in *gli1* expression in response to the SAG treatment. However, the increase in relative *gli1* expression was lower than that in the parental cell line (Fig. 3A). The relative levels of *ptch1* expression increased in WT clones to a degree similar to that of the ASEC-1 cell line (Fig. 3B). Conversely, *ift88* mutant clones #28 and #30 showed no change in *gli1* and *ptch1* expression in response to the SAG treatment (Fig. 3A,B).

In mice and zebrafish, the primary cilium also plays a role in repressing the Hh signaling pathway in the absence of ligand stimulation, leading to a higher level of unstimulated, basal Hh pathway activity in mutants lacking cilia (Huang and Schier, 2009; Huangfu et al., 2003). In zebrafish, the increase in unstimulated pathway activity in the absence of cilia is strong and mediated by unrestrained Gli1 activator (Huang and Schier, 2009). In mice, the increase in activity in the absence of cilia is weak and is mediated by partial loss of Gli3 repressor formation (Huangfu et al., 2003). To determine whether there is a difference in basal *gli1* expression levels between ASEC-1, WT clones, and *ift88* mutants, we analyzed *gli1* expression in these cell lines relative to the parental cell line in the absence of SAG. Relative to the parental cell line, we found that both mutant clones showed elevated basal expression of *gli1*, whereas basal expression in the two wild-type clones did not differ statistically from the parental line (Fig. 3C). Together, our data indicate that *ift88* mutant clones fail to induce the expression of Hh target genes in response to SAG and suggest that cilia are also required in anoles for repression of Hh pathway activity in the absence of inducer.

### Pharmacological induction of Hh signaling *in ovo*

**Fig. 4.**
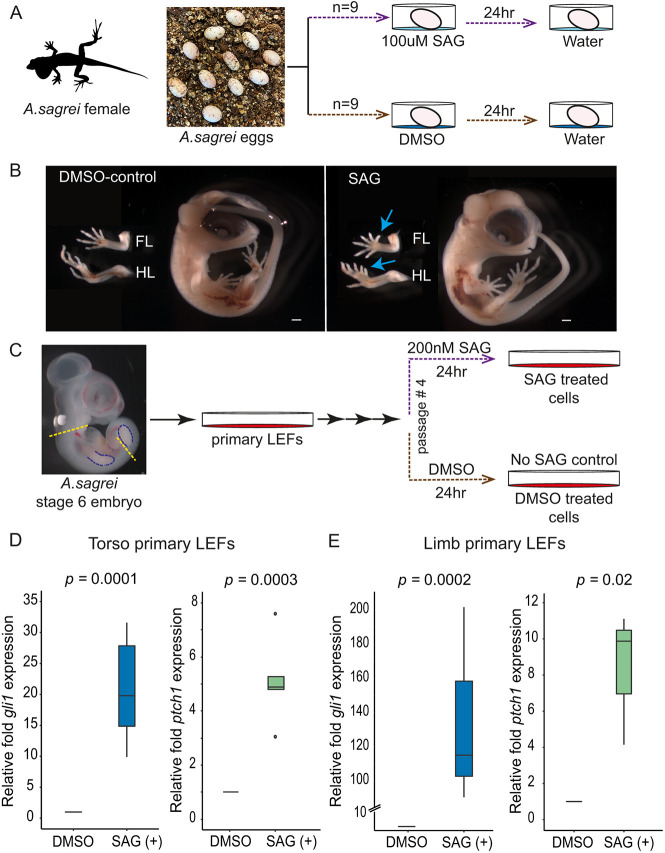
**Manipulation of Hh signaling in *A. sagrei* embryos and primary cells*.*** (A) Schematic of *in ovo* treatment of lizard embryos with SAG*.* Each egg was collected within 24 h of being laid and placed on top of a SAG or DMSO solution. These solutions were replaced by water after 24 h. (B) Embryos were collected from SAG- or DMSO-treated eggs after 14 days of incubation. Blue arrows highlight the location of extra digits. FL, forelimbs; HL, hindlimbs. Scale bar: 500 µm. (C) Schematic of primary lizard embryonic fibroblast cell (LEF) SAG treatment. Cells from either the torso (region between dashed yellow lines) or limb buds (outlined in blue) from a single embryo were grown in culture before being treated with SAG or DMSO. (D,E) qRT-PCR of *gli1* (blue) and *ptch1* (green) in the primary torso (*n*=5) (D) or limb (*n*=3). (E) LEFs exposed to 200 nM SAG or DMSO for 24 h. Expression was normalized to *gapdh* (paired *t*-test). The box limits represent lower and upper quartile values of the dataset. The horizontal line represents the median value of the dataset. Whiskers mark the minimum and maximum value of the data points in the given dataset.

Our ASEC-1 experiments tested whether the requirement of primary cilia function for SAG-induced Hh signaling in anoles is conserved. To validate the use of SAG for Hh pathway induction *in vivo* and to complement our studies in ASEC-1, we determined whether the developmental response of *Anolis* embryos to SAG exposure is also conserved. Previous work has demonstrated that a single injection of SAG into pregnant mice at embryonic day (E) 9.25 or E10.5 is sufficient to activate Hh signaling in the limb buds and induce polydactyly (Fish et al., 2017; Shin et al., 2019). To test our ability to pharmacologically manipulate Hh signaling in anoles, we exposed *Anolis* eggs to a single bolus of SAG within 24 h of oviposition (Fig. 4A; see Materials and Methods). Although it is not feasible to perform timed matings in anoles, *Anolis* embryos within these 24-h eggs are primarily at Sanger stages 3-4, which are at early stages of limb bud outgrowth and are roughly equivalent to E9.5-E10 mouse embryos (Sanger et al., 2008). However, we note that embryos in 24-h eggs will occasionally be at earlier or later stages of development. To assess whether SAG exposure can induce polydactyly in anoles, SAG-exposed eggs were incubated for 15 days to allow digits to develop. Polydactylous embryos were observed in eight out of nine eggs treated with 100 µM SAG, with four embryos exhibiting polydactyly in forelimbs and hindlimbs and four embryos displaying polydactyly in only the hindlimbs (Fig. 4B; Table S3). The single SAG-treated embryo that did not show polydactyly was at a later stage of development (stage 16 versus stage 11-14 for the polydactylous embryos), suggesting that the embryo was likely beyond stage 3-4 at the time of SAG exposure. None of the control embryos treated with vehicle (DMSO) alone was polydactylous. Our results demonstrate that, as in mice, SAG can induce polydactyly in anoles.

### Comparison of transcriptional responses in *A. sagrei* and *Mus musculus* after Hh pathway induction

The *in ovo* induction of polydactyly by SAG is consistent with the activation of Hh signaling. To confirm that SAG induces Hh transcriptional responses in anole primary cells, we isolated LEFs from torsos (*n*=5) and limbs (*n*=3) of late 5/early stage 6 *Anolis* embryos (Fig. 4C). Upon SAG exposure, *gli1* and *ptch1* were reproducibly induced in primary LEFs from the torso region and from limb buds. The average induction of *gli1* and *ptch*1 in torso LEFs was approximately 20-fold and 5-fold, respectively (Fig. 4D). The induction of these genes in response to SAG was also observed in limb LEFs, with *gli1* and *ptch1* increasing by 136-fold and 8-fold, respectively (Fig. 4E). Having established that SAG can induce the expression of two well-conserved transcriptional targets of the Hh pathway in *Anolis* primary cells, we next sought to determine the extent to which Hh induced transcriptional responses are shared between *A. sagrei* and *Mus musculus*.

**Fig. 5.**
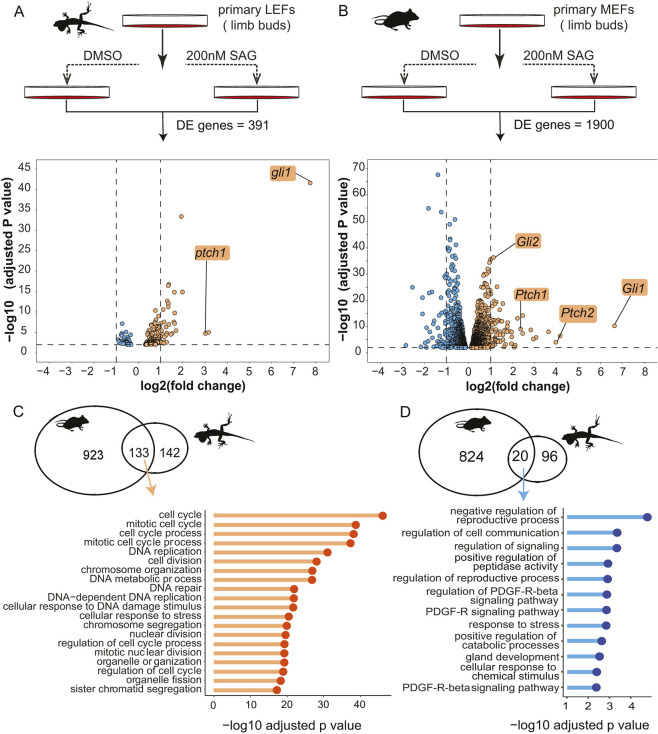
**SAG-responsive genes in *Anolis* and mouse primary LEF cells.** (A,B) Experimental design and volcano plots of differentially expressed (DE) genes in *Anolis* (A) and mouse (B) cells*.* Volcano plots display DE genes with an adjusted *P*<0.05 for *Anolis* and *P*<0.01 for mouse. Orange indicates upregulated and blue downregulated genes in response to SAG. (C,D) Venn diagrams present the number of shared and species-specific upregulated (C) or downregulated (D) genes in *Anolis* and mouse*.* Lollipop plots show the top biological processes GO terms associated with the shared upregulated or downregulated genes.

To compare Hh-induced transcriptional changes in anoles and mice, we stimulated the Hh signaling pathway in primary limb LEFs and primary limb mouse embryonic fibroblasts (MEFs) with SAG. Based on limb bud development, E11.5 mouse embryos are roughly equivalent to *Anolis* embryos at the late 5 to early 6 Sanger stages (Sanger et al., 2008). Primary LEFs and MEFs were collected from stage-matched embryos to generate parallel datasets from these two distantly related species. Each biological replicate of LEFs or MEFs was collected from all four limbs of a single embryo. Culture conditions for the two species were identical except for the temperature, with LEFs being grown at 29°C and MEFs at 37°C. After exposing cells to 200 nM SAG or vehicle (DMSO) alone for 24 h, RNA was isolated and sequenced. After performing RNA-seq, differentially expressed genes between SAG and DMSO treated cells were identified (Fig. 5A,B). We noted that there was more variation in gene expression across the lizard biological replicates than in the mouse replicates (Fig. S3; Bartlett's test of equal variance, *P*<2.2×10^−16^). Possible reasons for the greater variability in the *Anolis* data include a greater spread in developmental stages or higher levels of genetic variation in the *Anolis* embryos used; the mouse embryos were collected from a single litter whereas each *Anolis* embryo came from a separate egg laid by a different female. Because of this variability, we used a less-stringent significance threshold when identifying SAG responsive genes in lizard than mouse (adjusted *P*<0.05 for lizard versus *P*<0.01 for mouse). We found that 275 genes were significantly upregulated, and 116 genes were downregulated in response to SAG treatment in *Anolis* cells. In mouse, 1056 genes were upregulated, and 844 genes were downregulated in response to SAG.

Rather than using an arbitrary fold-difference threshold to determine which genes are likely to be biologically relevant Hh-responsive genes, we focused on genes that were SAG responsive in both anole and mouse. A comparison of lizard and mouse SAG responsive genes revealed a total of 181 shared genes (Fig. 5C,D; Table S4). Of these shared SAG responsive genes, 73% (133 genes) were upregulated in both species and 11% (20 genes) were downregulated in both species. Thus, the majority (84%) of shared SAG responsive genes have a transcriptional response in the same direction in both species. Included among these shared genes are *Gli1*, *Ptch1*, and *Ptch2*, which are all upregulated in limb LEFs and MEFs in response to SAG and are known direct transcriptional targets of Hh signaling. Similarly, *Hip1* was downregulated in both species upon SAG treatment. Further gene ontology (GO) enrichment analysis of the shared upregulated genes demonstrated that this group is enriched for genes associated with cell cycle regulation (Fig. 5C). A small number of genes responded in opposite directions and were either upregulated in *Anolis* cells yet downregulated in mouse cells (7%) or downregulated in *Anolis* but upregulated in mouse cells (8%). Other genes with different SAG responses include *Gli2*, which is a major activator of the Hh signaling pathway in mice (Bai et al., 2002; Ding et al., 1998; Mo et al., 1997). *Gli2* was upregulated in SAG-treated MEFs but was not responsive in LEFs. Similarly, *Gas1* and *Boc* were downregulated in mouse but did not exhibit a SAG response in *Anolis* cells.

## DISCUSSION

Squamates display tremendous diversity in a host of anatomical traits, such as limb length, digit morphology, degree of body elongation, and pigmentation (Simões and Pyron, 2021; Kuriyama et al., 2020). However, mechanistic studies that use functional genetic tools to understand developmental and cellular processes in this species-rich group are lacking. A major barrier has been the absence of effective, inexpensive embryo and gene manipulation tools, methods, and reagents. Encouragingly, *in vivo* gene-editing methods have recently been established in three different squamate models (Abe et al., 2023; Rasys et al., 2019; Tzika et al., 2023). Nevertheless, the field of squamate developmental genetics is still new and remains underdeveloped. The aim of the current study was to develop new resources for *A. sagrei* to further advance this species as a reptilian model system for comparative evolutionary, developmental and functional genetic studies. Specifically, we generated a new *Anolis* embryonic cell line and demonstrated that the line can be used for *in vitro* studies of gene function by using CRISPR/Cas9 gene editing of the *ift88* gene to investigate the role of the primary cilium in Hh signaling. In parallel with the generation of the ASEC-1 cell line, we refined *in ovo* methodologies and produced transcriptome resources to advance studies of Hh signaling in *A. sagrei*.

### Establishment of a new cell line for studies of reptilian gene function

To explore developmental mechanisms, gene function, and gene regulation in squamates, a reptilian model system with effective gene manipulation tools is essential. To complement *in vivo* gene-editing methods in *A. sagrei* (Garcia-Elfring et al., 2023; Rasys et al., 2019), we developed a resource for functional genetic experiments in cultured fibroblasts to increase the versatility of *A. sagrei* as a reptilian model system. The use of cell culture is ideal for studies that require fine control of the cellular environment and for studies investigating processes occurring at the cellular level (e.g. organelle biogenesis). Cell culture also provides the opportunity to perform high-throughput screens of gene function and is ideal for pharmaceutical manipulation of cells.

Although the ability to culture and manipulate primary cells is important, immortalized lines provide the ability to perform well-controlled studies on genetically homogeneous cell populations. We created an immortalized fibroblast cell line, ASEC-1, by serially passaging *A. sagrei* torso LEFs and isolating clones that escaped senescence (Fig. 1A). Our results demonstrate that ASEC-1 cells can induce Hh signaling in response to SAG and that CRISPR/Cas9-mediated methods can be effectively used to perform gene editing in this cell line. The ASEC-1 transcriptome datasets that we generated provide a deeper characterization of the cell line and are a valuable resource for other researchers who wish to use this line.

Based on their expression profile, we suggest that ASEC-1 cells are best described as myofibroblasts as they show properties of both definitive fibroblasts and mature mural cells (vascular smooth muscle cells/pericytes). Although fibroblasts and mural cells show similarities, gene expression profiling in mammals has helped to identify distinguishing features that vary between these cell types (Muhl et al., 2020). Myofibroblasts show features of both cell types. As one example, *vimentin* is a marker expressed by both mural cells and fibroblasts and is expressed very strongly in ASEC-1 cells. However, in some respects, ASEC-1 cells resemble adult fibroblasts more than they do mural cells. ASEC-1 cells express moderate levels of *pdgfra*. This gene is expressed in most fibroblast subtypes but it is not expressed in mural cell types. Moreover, ASEC-1 cells express high levels of *col1a1*, *col1a2*, and *lumican*; these three genes are expressed strongly in most mammalian fibroblast subtypes but are expressed very weakly in mural cell types. Yet in other ways, ASEC-1 cells resemble mural cells and myofibroblasts more than definitive fibroblasts. *Des*, *acta2*, *myo9*, and *cald1* are considered to be markers of myofibroblasts (Grigorieva et al., 2023)*.* These genes are highly expressed in ASEC-1 and mural cell types but are expressed weakly in definitive fibroblasts. Moreover, *Notch3* and *Mcam* display little expression in fibroblasts but both are highly expressed in both mural cells and ASEC-1 cells. Taken together, we suggest that ASEC-1 cells most closely resemble mammalian myofibroblasts, but given the large evolutionary distance between lizards and mammals, we view this conclusion as tentative.

As is typical for immortalized cell lines (Rebuzzini et al., 2016), ASEC-1 carries a small number of large duplications and deletions. We have defined these chromosomal anomalies to allow researchers to account for these changes when planning experiments. An important additional consideration when using ASEC-1 is that the cell line is derived from wild-caught animals from an invasive population in Florida. Since invasive populations of *A. sagrei* have high levels of genetic variation (Bock et al., 2021), we performed whole-genome sequencing (WGS) on ASEC-1 to annotate the set of polymorphisms present within the cell line. When selecting CRISPR target sites or designing primers, awareness of these polymorphisms is important, and our annotations will facilitate future gene-editing experiments in ASEC-1.

### Understanding the role of primary cilia in reptile Hh signal transduction

Some aspects of Hh signal transduction appear to be changing over vertebrate evolution, but detailed studies of Hh signaling have only been performed in a few model species, preventing a deeper understanding of when and how changes in Hh signaling mechanisms have evolved in vertebrates. Evolutionary reduction in limb length and limb loss in snakes has been associated with the progressive loss of function of a limb-specific cis-regulatory element of the *Shh* gene (Kvon et al., 2016; Leal and Cohn, 2016). These studies highlight the importance of Hh in reptile development and evolution and demonstrate how the expression of Hh signaling components can change across time. However, mechanistic investigations are needed to understand the details of how Hh signaling is transduced in reptiles and the extent to which transcriptional responses are shared between reptiles and more distantly related species.

While this study serves as proof of principle that the ASEC-1 cell line can be used to test the role of lizard genes in cellular processes, it also provides the first insights into Hh signal transduction in reptiles. The *ift88* gene is deeply conserved and is essential for the formation of cilia in species ranging from *Chlamydomonas* to mice (Huangfu et al., 2003; Pazour et al., 2000). In addition, primary cilia are known to be essential for Hh signal transduction in diverse vertebrates, including mouse, chick, and zebrafish (Ben et al., 2011; Huang and Schier, 2009; Huangfu et al., 2003; Huangfu and Anderson, 2005; Park et al., 2006; Yin et al., 2009). Our demonstration that disruption of *ift88* in lizard cells results in the loss of primary cilia and our conclusion that primary cilia are required for Hh signaling in lizards further support the broad conservation of these roles across vertebrates.

Mutant mouse embryos lacking cilia show a pronounced loss of ligand-induced Hh pathway activation (Huangfu et al., 2003; Jia et al., 2009). In these mutants, cilia-dependent Gli2 activation is lost, which in turn dramatically suppresses *Gli1* expression. The formation of Gli3 repressor is also greatly reduced in such mutants (Huangfu and Anderson, 2005). These mutants show some mild, ligand-independent (derepressed) activity in the neural tube due to reduced Gli3 repressor (Huangfu et al., 2003). Chicken Talpid3 mutants lacking cilia show dramatic loss of expression of Hh target genes in the neural tube, accompanied by ectopic ligand-independent expression of some Hh targets in the limbs (Yin et al., 2009). The derepression of some targets is likely explained by the reduced ability to generate GLI3 repressor (Davey et al., 2006). In contrast, zebrafish lacking cilia appear to show stronger ligand independent Hh pathway derepression, which cannot be further increased by Hh ligands (Huang and Schier, 2009). The derepression of the pathway in zebrafish *ift88/oval* mutants seems to be mediated by Hh-independent activation of Gli1 protein, rather than by a reduction in Gli repressor activity (Huang and Schier, 2009; Karlstrom et al., 2003). We observed a 3- to 4-fold increase in basal *gli1* expression in unstimulated *ift88* LEFs relative to wild type (Fig. 3C), whereas in *Ift88* mutant MEFs (without stimulation) derepression of the Hh pathway was not observed (Ocbina et al., 2009). Thus, it appears that in squamates, similar to ray-finned fish and birds, cilia may play a more important role in ligand-independent repression of the Hh pathway relative to mammals. We speculate that cilia in the shared vertebrate ancestor played an important role in Hh pathway repression, as well as in ligand-induced activation. However, the repressive role of cilia may have become less important in the mammalian lineage after its divergence from reptiles.

### Developmental and transcriptional responses to Hh activation in lizards and mice

Functional analysis of Hh signaling in squamates not only provides an opportunity to understand the role of Hh in reptilian development and regeneration but also can provide an evolutionary perspective on the mechanisms, regulation, and components of the Hh pathway. In parallel with our ASEC-1 experiments, we sought to expand methods and transcriptome resources for studies of Hh in reptiles. To induce Hh signaling in developing embryos, we optimized a method for the uptake of the pharmaceutical compound SAG into eggs by absorption. Hh signaling is known to regulate digit patterning and identity. For instance, polydactyly occurs in several naturally occurring mouse mutants and knockout mouse models as a result of increased activation of Hh signaling in the developing limb buds (Anderson et al., 2012; Hill et al., 2003; Lettice, 2003; Lettice et al., 2012; Tickle et al., 1975; Tickle and Towers, 2017). We have found that SAG exposure reliably induces polydactyly in anoles as observed in mice (Fig. 4B; Fish et al., 2017; Shin et al., 2019). The specificity and reproducibility of SAG-induced changes in digit number in anole embryos is consistent with hyperactivation of Hh signaling. We speculate that the variation in the number of digits in the forelimb and hindlimb is likely attributable to variation in embryonic stages in 24-h laid eggs and biological availability of SAG to the developing embryo. In related work reported by Sanger and colleagues, Hh signaling in anoles was manipulated by soaking eggs in cyclopamine, a Hh signaling inhibitor (Sanger et al., 2021). The robustness, simplicity, and cost-effectiveness of *in ovo* exposure to pharmaceutical agents provides a useful tool for the study of Hh signaling at specific embryonic stages. Although the absorption of other bioactive compounds into the egg remains to be tested, this general approach may also provide an effective way to perturb other signaling pathways and complements functional analyses based on the generation of constitutive gene knockouts in anoles.

Hh transcriptional responses have been explored in many different developmental contexts and in different model systems (Goodrich et al., 1996; Ingham et al., 2011; Sigafoos et al., 2021). Through these studies, we know that there are a small set of genes that consistently exhibit transcriptional responses to Hh across different tissues and different animal models. Among this core set of Hh responsive genes are components of the Hh signaling pathway itself, including genes that encode PTCH and GLI proteins (Anderson et al., 2012; Chiang et al., 1996; Ding et al., 1998; Holtz et al., 2013; Hu and Helms, 1999; Karlstrom et al., 1999; Lei et al., 2004; Matise et al., 1998; Mo et al., 1997; Nüsslein-Volhard and Wieschaus, 1980; Sasaki et al., 1997; Shimeld et al., 2007). However, a large proportion of Hh transcriptional responses are cell type specific. Although it has been demonstrated that some cell type-specific Hh responses are conserved across species (Bai et al., 2004; Briscoe and Small, 2015; Krauss et al., 1993; Patten et al., 2003; Persson et al., 2002; Ren et al., 2020; Ribes et al., 2010; Stamataki et al., 2005), comprehensive comparisons of Hh-induced transcriptional changes in homologous cell populations from different species are largely absent from the literature.

Here, we performed parallel investigations of Hh-induced transcriptional changes in mice and lizards in which the tissue source (limb buds), developmental stage, culture conditions, and treatment regimen were carefully controlled. The result is a unique transcriptional resource for the comparison of transcription patterns in homologous primary cells from two distantly related amniotes. *Anolis* lizards and mice diverged from a common ancestor more than 300 million years ago (Hedges et al., 2006), but share similar pentadactyl digit patterns. Moreover, in both species hyperactivation of Hh signaling during limb bud stages induces polydactyly. Therefore, limbs are ideal for investigating how Hh-induced transcriptional changes have evolved across time. We determined that the mouse orthologs of nearly half of the Hh-responsive lizard genes also exhibit Hh-induced transcriptional changes. Furthermore, the majority of these shared Hh-responsive genes (84%) have transcriptional shifts in the same direction in the two species. Thus, our comparison of Hh-responsive genes in limb cells from these species has allowed us to reveal the degree to which Hh signaling induces conserved transcriptional responses. Species-specific differences in Hh transcriptional responses may be equally important in understanding how the pathway regulates animal development. The Hh pathway induces transcriptional responses through GLI transcription factors. Upon induction of Hh signaling, GLI proteins can be activated through post-translational processing as well as through transcriptional upregulation of *Gli* genes. These two mechanisms act to shift the balance of Gli^Act^ to Gli^Rep^ (Falkenstein and Vokes, 2014). It is evident from studies in zebrafish and mice that the relative importance of the different Gli genes can evolve, and the details of how the ratio of Gli^Act^ to Gli^Rep^ is controlled can differ between species or between cell types in an organism (Bai et al., 2002; Huang and Schier, 2009; Karlstrom et al., 2003; Matise et al., 1998; Mo et al., 1997; Park et al., 2000; Tyurina et al., 2005). While *gli1* transcription is strongly induced by SAG in both mouse and lizard limb cells, we found that *gli2* is induced by SAG only in mice. These results suggest that *gli2* transcriptional regulation by Hh signaling differs between lizards and mice. However, whether the relative roles of the different Gli genes differ between mice and lizards will require additional functional studies in lizards.

### Conclusion

Although we have used ASEC-1 to explore Hh signaling here, this cell line can be used to broadly explore gene function in anoles. We note that Zhang and colleagues were able to isolate a subclone of ASEC-1 that can be induced to initiate myogenesis in culture (Zhang et al., 2022). They subsequently used this subclone to investigate the function of the *mymk* gene in lizard myoblasts. As lizards are poikilothermic, the immortalized *A. sagrei* cell lines grow at lower temperatures than mammalian cell lines (29°C versus 37°C). Therefore, these lines also allow for genetic investigation of homeostatic regulation of cellular processes at lower temperatures. This could be particularly useful for researchers studying the biology of thermoregulation. Moreover, since ASEC-1 was derived from a male (XY) this line may prove useful in addressing questions related to the biology of *Anolis* sex chromosomes and X chromosome dosage compensation mechanisms. For investigators who wish to perform comparative studies of gene function in reptiles, this cell line provides an easily accessible, inexpensive, and renewable resource.

## MATERIALS AND METHODS

### Animals

*Anolis sagrei* captured in Orlando (FL, USA) were housed at the University of Georgia following published guidelines (Sanger et al., 2008). Breeding cages housed up to five females and one male together, and nest boxes were checked daily to collect eggs within 24 h of being laid. Outbred ICR background (Envigo) mice were used for collecting MEFs for RNA-seq experiments. All experiments followed the National Research Council's Guide for the Care and Use of Laboratory Animals and were performed with the approval and oversight of the University of Georgia Institutional Animal Care and Use Committee.

### Induction of Hh signaling via *in ovo* SAG treatment

A total of 18 eggs were collected within 24 h of being laid. The eggs were carefully cleaned with Kimwipes to remove any debris. In a 24-well plate, each egg was carefully placed in an individual well. A 10 mM SAG stock solution in 100% DMSO solvent was prepared (CAS 912545-86-9, Cayman Chemical Company). Preliminary experiments indicated that treatment of eggs with a single bolus of 100 µM SAG or higher was sufficient to consistently induce changes in digit number. A 100 µM SAG solution from 10 mM SAG stock was prepared using water as solvent. A control solution with an equivalent volume and concentration of DMSO was prepared. The final concentration of DMSO in the control solution was 1%. In each well, 80 µl of either 100 µM of SAG or control DMSO solution was placed below the eggs carefully, ensuring the egg was in contact with the liquid (Fig. 4A). Nine eggs received 100 µM of SAG solution and nine received control DMSO solution. Eggs were incubated at 29°C. After 24 h, SAG and DMSO solutions were replaced by 80 µl of water. The eggs were incubated for 14 days at 29°C. The eggs were monitored daily and 80 µl of water was added as and when required to avoid drying the eggs. On the 15th day, eggs were dissected in 1× PBS solution, and embryos were examined for morphological differences. Developmental staging was performed according to Sanger et al. (2008). Embryos were imaged and fixed in 4% paraformaldehyde (PFA) before being dehydrated in a methanol series, starting from 25% and ending in 100%. Embryos were stored in 100% methanol at 4°C.

### Culturing of primary LEFs

*A. sagrei* eggs were cleaned with Kimwipes soaked in 70% ethanol. All debris on the eggshell was removed, and eggs were given two or three quick washes with 1× PBS to clean the eggshell. The eggs were dissected in sterile 1× PBS (pH 7.4, Molecular Biology Grade, without Calcium and Magnesium, Corning, 21-040-CMR). Individual stage 6 embryos were transferred into a clean Petri dish with sterile 1× PBS containing 1× Penicillin-Streptomycin-Amphotericin B solution (Corning, 30-004-CI). The embryo was gently washed by swirling the dish. The process was repeated at least three times. For the isolation of LEFs from limb tissue, using sterile forceps, limb buds were separated from the embryo. All four limb buds were aseptically transferred in a 1.5 ml microcentrifuge tube with 0.5 ml 0.05% Trypsin-EDTA (Corning, 25-051-CI). For collecting LEFs from the torso region (Fig. 4C), the head, tail, and limb buds were removed with sterile forceps. The rest of the tissue was eviscerated. The tissue was then teased with the forceps and aseptically transferred into a 1.5 ml microcentrifuge tube with 0.5 ml 0.05% Trypsin-EDTA. The tissue was then incubated at 29°C for 45-60 min. To facilitate tissue disaggregation, the tissue was teased by pipetting the trypsin solution up and down gently at 15 min intervals. After the incubation, the trypsin was neutralized by adding an equivalent volume of LEF growth medium [1× DMEM with 4.5 g/l glucose, without L-glutamine and sodium pyruvate (Corning 15-017-CM), 10% heat-inactivated fetal bovine serum (GeminiBio, Benchmark, 100-106), 1× glutamine (Corning, 25-015-CI), 1× Penicillin/Streptomycin/Amphotericin B solution (Corning, 30-004-CI)]. Cells were collected by centrifuging at 1200-1300 rpm (500 ***g***) at room temperature (RT) for 5 min. Cell pellets were resuspended in 100 µl of LEF growth medium before being uniformly plated in a single well of a 24-well plate and incubated at 29°C at 5% CO_2_ for 1-1.5 h. This ensured that the cells were attached to the plate. Afterwards, 1 ml of LEF growth medium was added to each well, and cells were allowed to grow until they became confluent, which required around 48-72 h. This stage was designated passage 0. When the cells became confluent, they were trypsinized using 0.05% Trypsin-EDTA (pre-warmed to 37°C) to detach cells from the plate. Cells were incubated at 29°C for 1-2 min. The cells were observed under the microscope for detachment; if the cells did not detach from the plate, they were further incubated at 29°C for a few more minutes monitoring for dissociation. Gentle tapping of the cell culture vessel was used to promote detachment. When >90% of cells were detached, trypsin was neutralized by adding LEF growth medium. Cells were collected by centrifuging at 1200-1300 rpm (500 ***g***) at RT for 5 min. Cell pellets were resuspended in LEF growth medium plated in a single well of a 12-well plate and incubated at 29°C at 5% CO_2_. This was passage 1. Cells from an individual embryo were considered as one biological replicate. The cells were grown consistently at 29°C at 5% CO_2_.

### Pharmacological manipulation of Hh signaling pathway in primary LEFs

Primary limb and torso LEFs were collected from individual *A. sagrei* stage 6 embryos. Primary LEFs from an individual embryo were grown until passage 3 (primary limb LEFs ∼10-12 days; primary torso LEFs ∼7-8 days; counted from the initial collection of the primary cells from the embryo). Between the passages, the cells were split at a 1:2 ratio. After passage 3, the cells were split at a 1:2 ratio and seeded at equal density into two wells of a 24-well plate (passage 4). Primary limb LEFs required ∼3-4 days and primary torso LEFs required ∼2-3 days to become at least 70% confluent at each passage. After the passage 4 cells became confluent, they were serum starved for 48 h by adding serum starvation medium [1× DMEM with 4.5 g/l glucose, without L-glutamine and sodium pyruvate (Corning, 15-017-CM), 1% heat-inactivated fetal bovine serum (GeminiBio, Benchmark, 100-106), 1× glutamine (Corning, 25-015-CI), 1× Penicillin/Streptomycin/Amphotericin B solution (Corning, 30-004-CI)]. Serum starvation results in cell cycle exit inducing ciliogenesis (Pugacheva et al., 2007). Next, 200 nM SAG (prepared in serum starvation medium) was added to one well and a DMSO vehicle control solution (prepared in serum starvation medium) was added to the second well of each biological replicate; 24 h after the addition of SAG, RNA was collected. *gli1* and *ptch1* expression was quantified using qRT-PCR. The details for RNA isolation and qRT-PCR are described below.

### RNA-seq analysis of SAG responsive genes in *A. sagrei* and *M. musculus*

Five *A. sagrei* staged at late 5/early 6 (Sanger et al., 2008) were collected and limb buds were isolated by dissection. Primary limb LEFs were disaggregated as described above. We plated passage 0 cells in a 24-well plate. After 4-5 days, when the cells became confluent, cells were detached using trypsin and replated in a 6-well plate as described in methods for culturing primary LEFs. This was passage 1. Passage 2 cells were plated in a 60 mm plate and passage 3 cells were plated in a 10 cm plate. For passage 4, the cells were split into two wells in a 6-well plate. Primary limb LEFs required ∼8 days to become at least 70% confluent between the passages. Five embryos from *M. musculus* were collected at stage E11.5. Primary limb MEFs were collected in a similar fashion to the primary LEFs. MEFs were grown at 37°C at 5% CO_2_. Primary limb MEFs required 2-3 days to become at least 70% confluent between the passages.

Passage 4 limb LEFs and MEFs from individual embryos were divided into two wells; 200 nM SAG was added following serum starvation for 48 h to one well and the volume equivalent DMSO solution was added to the second well. The embryos were processed in a pair-wise manner and analyzed as paired sets. We generated five biological replicates for each treatment: primary LEFs treated with SAG, primary LEFs treated with DMSO control, primary MEFs treated with SAG, and primary MEFs treated with DMSO control. We collected total RNA 24 h after the SAG exposure using the mirVana miRNA Isolation Kit (Thermo Fisher Scientific). RNA-seq libraries were prepared using the TruSeq Stranded mRNA Library Prep Kit (Illumina) and were sequenced at the Georgia Genomics and Bioinformatics Core. After checking read quality with FastQC v.0.11.8, *A. sagrei* reads were aligned to the AnoSag2.1 genome reference (Geneva et al., 2022) and *M. musculus* reads were aligned to the mm10 genome using HISAT2 v.2.1.0. Transcripts were counted using the feature count function from Rsubread v.2.10.5 (Kim et al., 2019; Liao et al., 2019). Sample similarity and batch effects were assessed by principal component analysis. Bartlett's test for equal variance was applied to statistically test for differences between gene expression variation in data collected from the two species. Differentially expressed genes between SAG-treated and DMSO-treated samples in both species were identified using DESeq2 v.1.36.0. (Love et al., 2014). Batch effects due to sampling from different embryos were corrected by using biological replicates as the batch variable. Four candidate differentially expressed genes (*gli1*, *ptch1*, *cldn1* and *rmp2*) identified by RNA-seq analysis were validated by qRT-PCR in primary LEFs with three biological replicates (Table S5). GO enrichment analysis of upregulated and downregulated genes shared between *M. musculus* and *A. sagrei* was carried out using g: GOSt functional profiling, available in the g: Profiler program with 2019 update (Reimand et al., 2007; Raudvere et al., 2019). For the study, the ‘biological processes’ GO terms were analyzed.

### Generation of immortalized LEF cell lines

To generate immortalized *Anolis* cell lines, we adapted a method previously used for immortalization of MEFs (Xu, 2005). In brief, primary LEFs from the torso region of a single stage 6 *A. sagrei* embryo were collected as described above in the ‘Culturing or primary LEFs’ section. Passage 0 cells were plated in a 24-well plate. When the cells reached ∼80% confluency after 3-4 days, they were trypsinized and plated again in a 12-well plate (described in detail in the ‘Culturing or primary LEFs’ section). This was passage 1. Within 3-4 days the cells became confluent and were transitioned to a 6-well plate. This was considered passage 2. Passages 3, 4, and 5 cells were plated in a 60 mm plate with a 1:3 split ratio. At passage 6, the cells from a 60 mm plate were transitioned to a 10 cm plate. After the cells reached confluency on the 10 cm plate, we continued to serially passage the cells on 10 cm plates with a 1:3 split ratio until passage 24. After the 24th passage, we changed the split ratio to 1:6. The growth rate of the cells decreased at this time point as cells started undergoing senescence. At passage 26, very few cells were attached to the plate, and there were no obvious signs of cell growth for two weeks. This process took ∼4.5 months from the day we harvested primary LEFs. During this time, we frequently changed the LEF growth medium [1× DMEM with 4.5 g/l glucose, without L-glutamine and sodium pyruvate (Corning, 15-017-CM), 10% heat-inactivated fetal bovine serum (GeminiBio, Benchmark, 100-106), 1× glutamine (Corning, 25-015-CI), 1× Penicillin/Streptomycin/Amphotericin B solution (Corning, 30-004-CI)]. After this ‘no growth period’, visible cell growth was observed in the form of patches or colonies of cells. The few cells which escaped cellular senescence started growing at this point. We allowed these cells to grow and become confluent. Within 7-8 days of first observing patches of cell growth, the cells in the 10 cm plate were confluent. Cells were then trypsinized, and single cells were sorted using the MoFlo Astrios EQ (Beckman Coulter) cell sorter at the Cytometry Shared Resource Laboratory at the University of Georgia. Each cell was sorted into a well pre-filled with LEF growth medium in a 96-well plate. The cells were incubated at 29°C at 5% CO_2_. These single-cell clones started growing by ∼7 days and reached confluency within 15 days. Over a period of 30 days, we expanded these single cell clones from a 96-well plate to a 10 cm plate. The cells were grown consistently at 29°C at 5% CO_2_ and were typically passed at a ratio of 1:5 every 3 days. We then froze and stored individual lines in liquid nitrogen. The complete process of generating and expanding single-cell clones starting from the day we harvested the primary cells took ∼6 months.

### Morphology and doubling time of ASEC-1 cells

Overall ASEC-1 morphology was documented through brightfield imaging (phase contrast and differential interference contrast), which was performed on 4% PFA-fixed cells using an Olympus model CK40 and a Keyence BX-X710 microscope. ASEC-1 morphology was highlighted by fixing with 4% PFA and staining with DAPI (Sigma-Aldrich, D9542) and Alexa488-Phalloidin (Thermo Fisher Scientific, A12379). ASEC-1 doubling time was determined by plating cells in 24-well dishes and then harvested after trypsinization and counted using Trypan Blue (Sigma-Aldrich, T8154) staining and a hemocytometer (four wells per time point), first at 6 h after plating (t=0), and then every 24 h afterward for a total of 144 h. A regression fit was applied to the exponential growth portion of the series and the resulting equation used to determine doubling time (Fig. S2E).

### Freezing/thawing and density at confluency of ASEC-1 cells

For long-term storage of cells, we followed the same general protocol used for freezing mammalian cells. Cells were plated on a 60 mm or 10 cm plate and were allowed to reach at least 80% confluency. The cells were then detached from the plate using 0.05% Trypsin EDTA and the cell pellet was collected as described before. The cell pellet was uniformly resuspended in freshly prepared LEF freezing medium [10% DMSO +15% heat inactivated fetal bovine serum (GeminiBio, Benchmark, 100-106) +75% LEF growth medium]. At least 1,000,000 cells/ml were transferred into 1-2 ml cryovials. The vials were then stored for 24 h at −80°C in a Thermo Scientific Mr. Frosty freezing container before being transferred into liquid nitrogen for long-term storage. To thaw cells, we removed the cryovials from the liquid nitrogen storage and placed them into a 37°C water bath until about 80% defrosted. We then added 2 ml of pre-warmed (37°C) LEF growth medium into the vial and transferred the cell suspension into a 15 ml conical tube. Cells were collected by centrifuging at 1200-1300 rpm (500 ***g***) at RT for 5 min, and the cell pellet was resuspended in LEF growth medium before being plated on a 60 mm plate. The cells were then transferred into the incubator and grown at 29°C at 5% CO_2_. Survival after freezing was determined by harvesting growing cells and counting as above, then freezing four aliquots of 1×10^6^ live cells at −80°C. After 7 days of freezing, the aliquots were thawed onto tissue culture dishes. Then, 12 h, after plating, adherent cells were harvested, and live cells were counted using Trypan Blue (Sigma-Aldrich, T8154) staining and a hemocytometer. We determined that ∼79% of cells remain viable after thawing (Fig. S2H). Cell density at 100% confluency was determined by plating cells at 5×10^4^/cm^2^ (*n*=4 wells) and allowing cells to grow for 6 days of growth (visually inspected to confirm confluency), then counting as described above (Fig. S2F).

### Transfection efficiency and puromycin sensitivity of ASEC-1 cells

To estimate general transfection efficiency, cells were transfected with a CAG-EGFP construct (Addgene plasmid #16664, deposited by Dr Fred Gage; Zhao et al., 2006) using Lipofectamine LTX Plus (Thermo Fisher Scientific), fixed after 48 h with 4% PFA, counterstained with DAPI (Sigma-Aldrich, D9542), and imaged at 20× on a Keyence BX-X710 microscope. Puromycin sensitivity was performed by plating cells at 5×10^4^ cells/cm^2^ and then changing media after 24 h with medium containing 0, 1, 3, 6, 12, and 24 µg/ml puromycin (InvivoGen, ant-pr-1). After 48 h of selection, cells were harvested by trypsinization and counted as described above.

### Immunostaining and detection of primary cilia

In preparation for immunostaining, lizard cells were plated onto a gelatin-coated coverslip in a 24-well plate. Once the cells were confluent, they were serum-starved for 48 h to promote ciliogenesis, as described above. After 48 h, the cells were washed with 1× PBS twice and fixed in EMS grade 4% PFA for 15 min at RT. The cells were then washed with 1× PBS three times. Cells were incubated at RT for 1 h in freshly made blocking solution (10% heat-inactivated goat serum with 0.1% Triton X-100 in 1× PBS). Cells were then washed with wash buffer (1% heat-inactivated goat serum with 0.1% Triton X-100 in 1× PBS) for 10 min at RT. The cells were incubated with primary antibodies (acetylated α-tubulin mouse monoclonal antibody, Sigma-Aldrich, T7451, 1:8000; and ift88 rabbit monoclonal antibody, Abcam, ab184566; 1:125) overnight at 4°C. The following day, cells were washed three times with wash buffer at RT. The cells were then incubated in the dark for 1 h at RT with anti-mouse cy3 (Jackson ImmunoResearch, 715-167-003; 1:125), anti-rabbit Alexa Fluor 488 (Invitrogen, A21206, 1:125), with DAPI as a nuclear stain (Sigma-Aldrich, 1 mg/ml). After 1 h of incubation with the secondary antibodies, we mounted the coverslips on a slide, and VECTASHIELD antifade mounting medium was applied to preserve fluorescence. Slides were stored at 4°C until imaging. The cells were observed and imaged with a Zeiss LSM 880 confocal microscope. Primary cilia were identified based on acetylated α-tubulin antibody staining, the position of the nucleus, and the microtubule organization center in context to a ciliary structure. The primary cilia were then confirmed by ift88 antibody staining. A total of 117 cells for ASEC-1, 150 cells for WT clone #12, 137 cells for WT clone #35, 112 cells for mutant clone #28, and 158 cells for mutant clone #30 were analyzed.

### WGS and SNP identification of ASEC-1 cell line

The NEBNext Ultra II FS DNA Library Prep Kit for Illumina was used to prepare libraries for WGS of the ASEC-1 cell lines. Libraries were sequenced by Novogene Corporation Inc. Scripts for DNA read alignment are available online at https://github.com/tryggvimcdonald/iLEF-analysis-scripts/tree/main/genome_assembly_scripts. Genome coverage per scaffold was calculated using the pileup.sh command from BBMap (Bushnell, 2014). This same command was also used to calculate the per-bin coverage with various bin sizes. From the coverage of the whole genome, three regions on scaffolds 3, 6, and 7 with deletion or duplication events were identified. The depth command from SAMtools was used to generate average coverage for these regions by dividing the sum of coverage values by the total number of sites. For SNP calling, duplicate reads were first removed using Picard (‘Picard Toolkit’ 2019). Next, the resultant file was indexed with SAMtools, and a pileup file was created. BCFtools (Li, 2011) was used to identify SNPs. SNPs from alignments of low quality were excluded. For detailed methods refer to Geneva et al. (2022). shinyCircos v.1.0 (Yu et al., 2018) was used for the generation of the Circos plot.

### CRISPR/Cas9 gene editing of ASEC-1 cells

An IDT gBlock with a human U6 promoter (Mali et al., 2013) followed by a gRNA directed against *ift88* target sequence (5′-GTTCTTGAACTGGATGTACC-3′) was designed. ASEC-1 cells were transfected with a Cas9 plasmid carrying puromycin resistance [pSpCas9(BB)-2A-Puro (PX459) V2.0, Addgene, #62988] and the gBlock in a 1:1 ratio (a total of 1 µg DNA) using Lipofectamine LTX Plus (Thermo Fisher Scientific). Forty-eight hours after the transfection, the cells were placed under puromycin selection at a concentration of 25 µg/ml for 24 h. We determined that degree of puromycin exposure is 100% lethal to wild-type ASEC-1 cells (Fig. S2). After 24 h, puromycin-resistant cells were allowed to grow and become confluent. Cells were then trypsinized, and single cells were sorted into a 96-well plate as described above. The cells were incubated at 29°C at 5% CO_2_. These single-cell clones started growing in ∼7 days. Once cells started growing, within a time span of 30-40 days we expanded the clones, froze them, and stored them in liquid nitrogen for later analysis.

Cell lines were screened for indel mutations by PCR amplifying the targeted *ift88* region with the primers listed in Table S6. PCR conditions were 95°C 2 min; 30 cycles of 95°C 30 s-58°C 30 s-72°C 30 s; 72°C 5 min for final extension. PAGE protocol was adapted from VanLeuven et al. (2018). We performed electrophoresis at 150 mV for 7 h. Following PAGE screening, Sanger sequencing was performed on the PCR products to precisely identify sequence alterations in each cell line. Multiplex PCR-based sex genotyping of the ASEC-1 cell line was performed using primers listed in Table S6. The PCR conditions were 95°C for 30 s for initial denaturation; 35 cycles of 95°C for 30 s; 58°C for 30 s; and 72°C for 30 s; 72°C for 2 min for the final extension.

### RNA isolation and qRT-PCR

Cells were lysed in 500 µl Invitrogen TRIzol reagent and total RNA was isolated according to the Invitrogen TRIzol reagent user guide. RNA yield was measured using Qubit™ RNA High Sensitivity assay kit. cDNA was synthesized using ProtoScript II First Strand cDNA Synthesis Kit (New England BioLabs). *gli1* and *ptch1* mRNA expression in the cells exposed to SAG were measured by qRT-PCR using a Roche LightCycler 480. LightCycler 480 SYBR Green I Master (Roche) was mixed with gene-specific primers and 10 ng of cDNA template for each individual reaction. The PCR conditions were as follows: pre-incubation at 95°C for 5 min followed by 45 amplification cycles of 95°C for 10 s, 58°C for 10 s, and 72°C for 10 s. For each biological replicate, three technical replicates were used. For primary LEFs ΔCt values were calculated by normalizing *gli1* and/or *ptch1* expression with *gapdh* as the reference gene. For the remaining experiments performed, *tbp* and *atp5f1d* were used as the reference genes.

The RNA-seq dataset was used to identify and select reference genes for qRT-PCR. Transcript levels of *gli1* and *ptch1* were comparable with those of *tbp* and *atp5f1d*. The RNA-seq data indicates that *tbp* and *atp5f1d* were stably expressed in DMSO- and SAG-treated LEF samples. The stable expression was defined as a coefficient of variance below 0.5 within the biological replicates. We then confirmed stable expression of these genes in ASEC-1 cell line by analyzing Ct values between biological replicates when exposed to SAG and DMSO treatments. When more than one reference gene was used as a normalizer, the geometric mean of ΔCt values with the individual normalizer gene was calculated. This geometric mean of ΔCt value was then further used for 2 - ΔΔCt calculations. Statistical analyses were performed on ΔCt values using a paired *t*-test (Fig. 1; Fig. 4; Fig. S1), Wilcoxon signed-rank test, the Kruskal–Wallis rank sum test, and Kruskal–Wallis multiple comparison test (Fig. 3).

### Pharmacological manipulation of the Hh signaling pathway in ASEC-1 cells

A 24-well plate was seeded with an equal density of the ASEC-1 cells. Each well was considered a biological replicate. Cells were prepared and serum starved as described above. Cells were treated with SAG concentrations ranging from 50 nM to 500 nM. Control wells were treated with 0.05% vehicle (DMSO). For every SAG concentration, three biological replicates were assayed, and RNA was collected after 24 h of SAG treatment. In separate experiments, we exposed the ASEC-1 cell line to 200 nM SAG for time intervals ranging from 6 to 72 h. For each time interval, an equivalent volume of vehicle (0.02% DMSO) was used as a control. These experiments were repeated twice. For testing the SAG responsiveness of WT and *ift88* subclones, three biological replicates were treated with 400 nM SAG, and three biological replicates were treated with 0.04% DMSO for 24 h. This experiment was repeated three times.

### RNA-seq analysis for differentially expressed genes in ASEC-1 cells after Hh pathway induction

Passage # 34 ASEC-1 cells were thawed and plated on a 60 mm plate. Once the cells were confluent, they were plated on a 10 cm plate. After confluency, the cells were split into a 1:8 ratio and plated onto 8×10 cm plates. When the cells were confluent, they were serum starved for 48 h as described in the ‘RNA-seq analysis of SAG responsive genes in *A. sagrei* and *M. musculus*’ section. Following serum starvation for 48 h, 400 nM SAG was added to 4×10 cm plates and the volume equivalent DMSO solution was added to the remaining plates. The samples were processed in a pair-wise manner and analyzed as paired sets. We generated four biological replicates for each treatment: ASEC-1 cells treated with SAG, ASEC-1 cells treated with DMSO control. We collected total RNA 48 h after the SAG exposure using the mirVana miRNA Isolation Kit (Thermo Fisher Scientific). RNA-seq libraries were prepared using the TruSeq Stranded mRNA Library Prep Kit (Illumina) and were sequenced at the Georgia Genomics and Bioinformatics Core at the University of Georgia. After checking read quality with FastQC v.0.11.8, *A. sagrei* reads were aligned to the AnoSag2.1 genome reference (Geneva et al., 2022). Transcripts were counted using the feature count function from Rsubread v.2.10.5 (Kim et al., 2019; Liao et al., 2019). Sample similarity and batch effects were assessed by principal component analysis. Differentially expressed genes between SAG-treated and DMSO-treated samples were identified using DESeq2 v.1.36.0 (Love et al., 2014).

### Scanning electron microscopy

In preparation for SEM, lizard cells were plated onto a gelatin-coated coverslip in a 24-well plate. Once the cells were confluent, they were serum-starved for 48 h to promote ciliogenesis. After 48 h, the cells were washed with 1× PBS thrice and fixed at room temperature in a 2% glutaraldehyde and 2% PFA solution prepared by Georgia Electron Microscopy core facility. The cells were then imaged by the Georgia Electron Microscopy core facility using a Thermo Fisher Scientific (FEI) Teneo, a field emission scanning electron microscope (FESEM).

## Supplementary Material



10.1242/develop.204275_sup1 Supplementary information

Table S1. Upregulated Hh responsive genes in ASEC-1 cell line after 400 nM SAG exposure for 48hr.

Table S4. Differentially expressed genes in response to 200 nM SAG exposure for 24 hr in *A. sagrei* and *M. musculus* primary limb cells.
