# Supplementary material for: A new cell culture resource for investigations of reptilian gene function
Source: Development. 2024 Nov 22;151(22):dev204275. doi: 10.1242/dev.204275 (PMC11607698; doi:10.1242/dev.204275)
Supplement: Supplementary information [file develop-151-204275-s1.pdf]

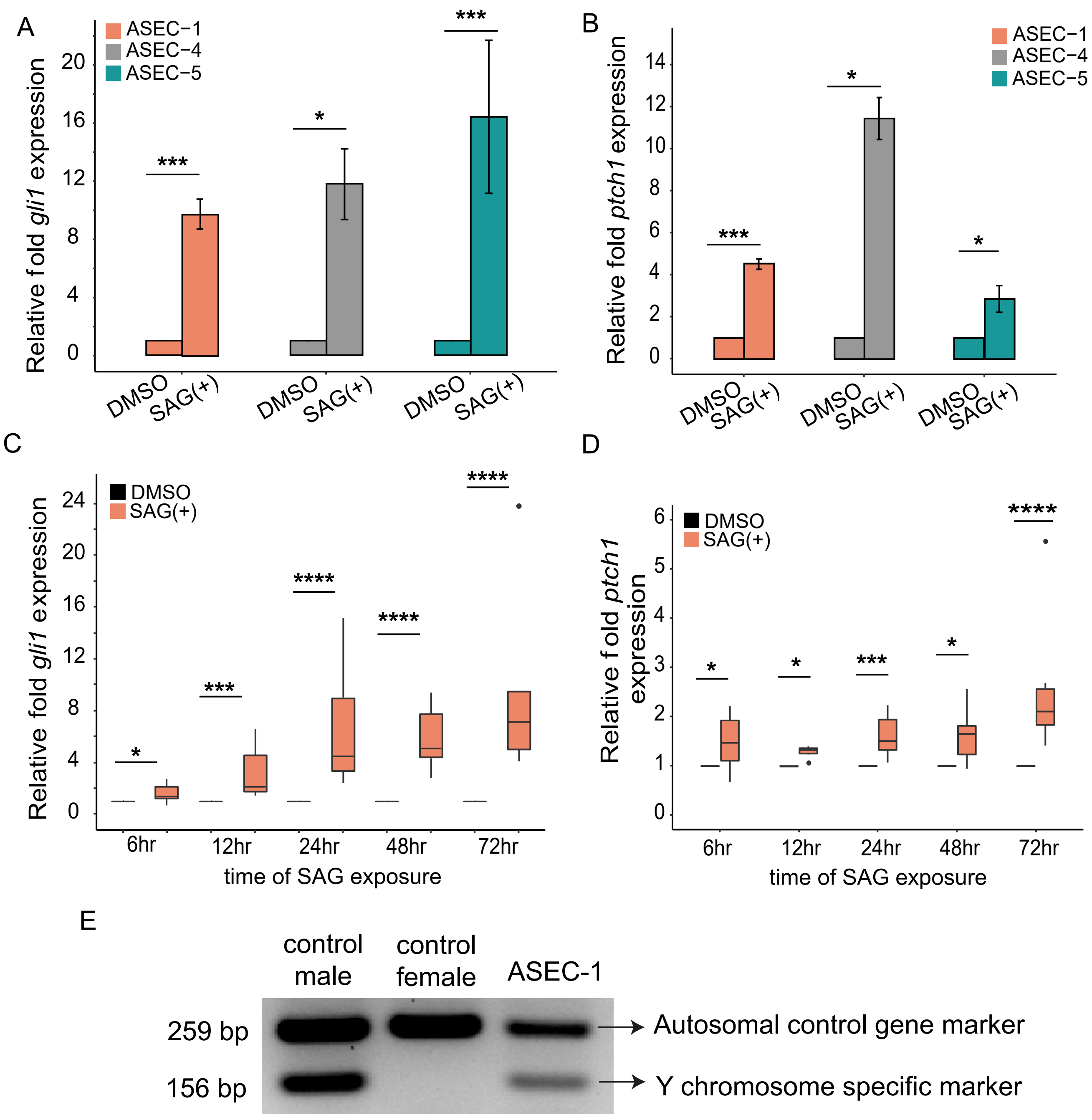

Fig. S1. SAG responsiveness of immortalized cell lines assayed by qRT-PCR and sex genotyping of ASEC-1.

(A-B) Relative fold *gli1* and *ptch1* expression in three immortalized clonal cell lines generated ASEC-1 (n=3), ASEC-4 (n=2), and ASEC-5 (n=3). The bar graph represents *gli1* and *ptch1* induction in SAG treated samples relative to DMSO control. The data was normalized to *gapdh* expression. For each biological replicate, there were three technical replicates. (C-D) Relative fold *gli1* and *ptch1* expression in the ASEC-1 cell line in response to 200 nM SAG exposure for different time intervals (n=6). The box plots represent *gli1* and *ptch1* induction in SAG treated samples relative to DMSO control for that time point. The data was normalized using two reference genes *tbp* and *atp5f1d*. * $p< 0.05$, ** $p<0.01$, *** $p<0.001$, **** $p<0.0001$. p values represent the statistical significance between delta Ct values of DMSO and SAG treated samples by paired t test (E) PCR genotyping results confirming the presence of Y chromosome in the ASEC-1 cell line.

Development • Supplementary information

Figure S2

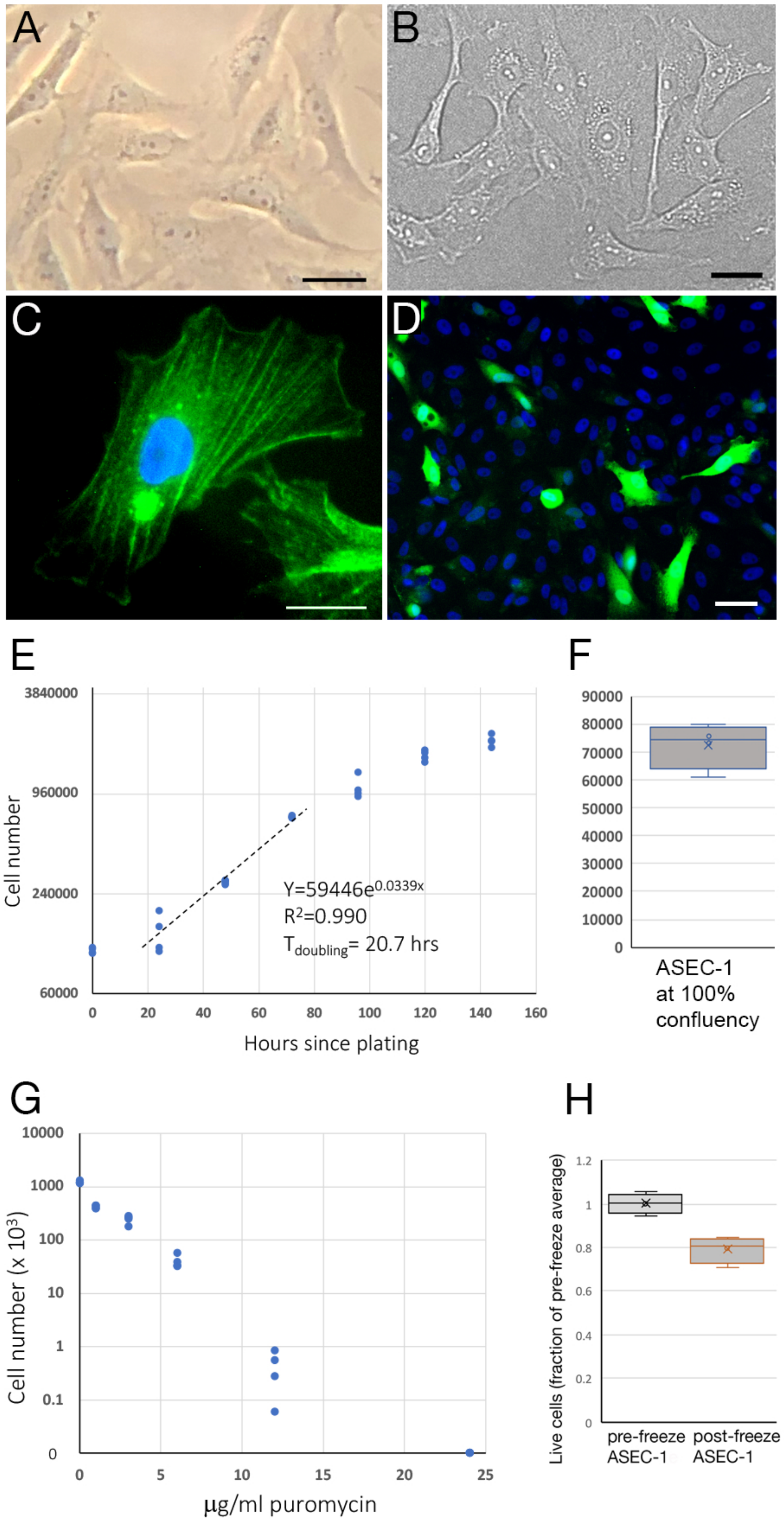

Development • Supplementary information

Fig. S2. Characterization of the ASEC-1 cell line.

ASEC-1 Lizard Embryonic Fibroblasts imaged by (A) phase contrast and (B) Differential Interference Contrast are shown. (C) An ASEC-1 cell stained with phalloidin and DAPI, showing typical fibroblast morphology. (D) ASEC-1 cells were transfected with a EGFP expression construct using Lipofectamine 3000 and imaged 72 hours later after DAPI staining. Transfection efficiency was estimated to be approximately 9%. (E) ASEC-1 doubling time during the exponential growth phase was calculated as 20.7 hours from an exponential regression fit. (F) The average cell density at 100% confluency was 72,825 +/- 8,243 cells/cm^2. (G) Puromycin dose sensitivity was determined after 48 hours exposure: ~99% and 100% of cells were killed at 12ug/ml and 24ug/ml puromycin, respectively. The fraction of cell surviving after 7 days freezing/thawing (h) was determined to be 0.79 +/-0.06. Scale bars in a-d represent 50 μm.

Development • Supplementary information

Figure S3

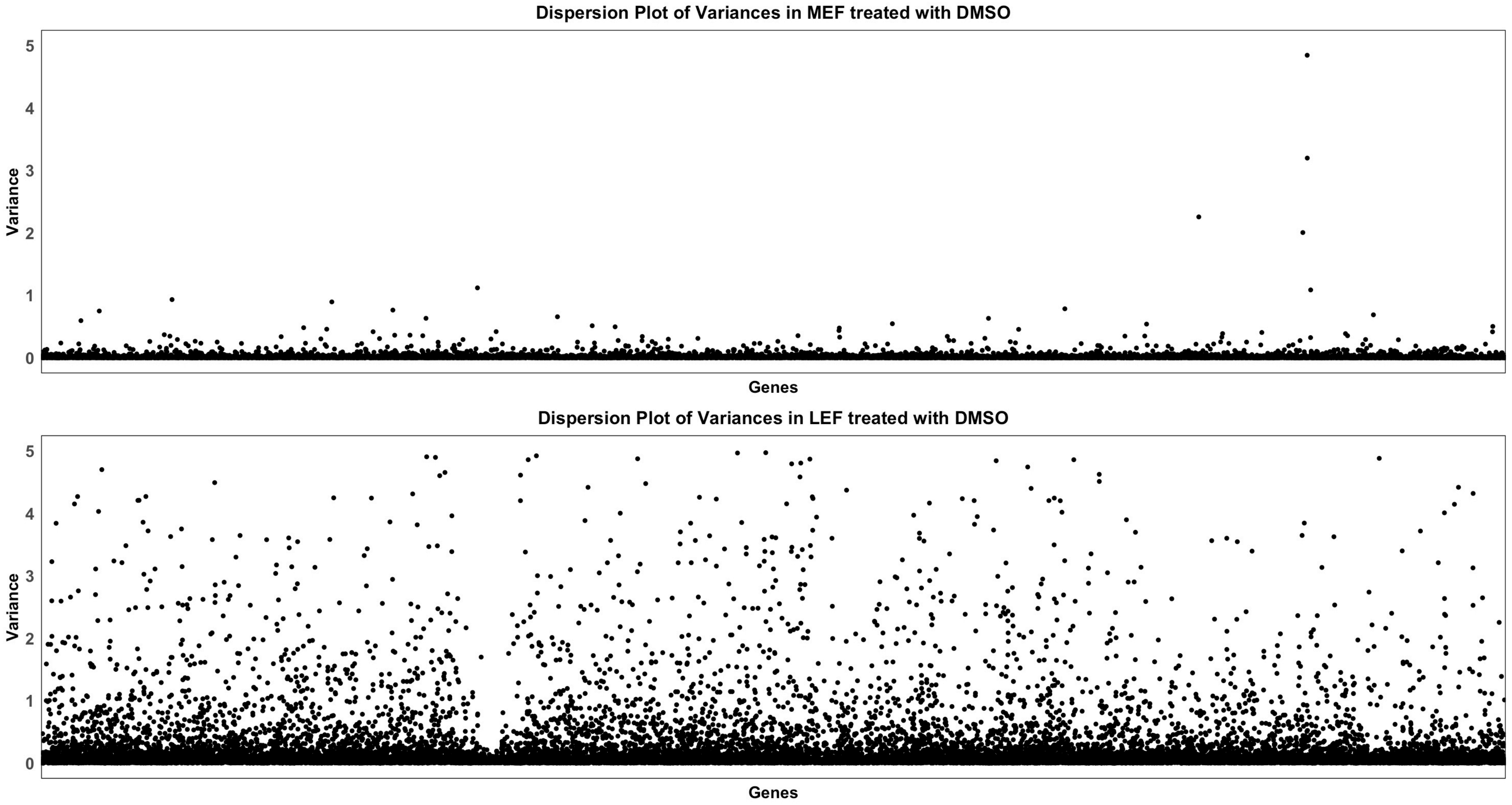

Fig. S3. Comparison of gene expression variance in MEF and LEF RNA-seq replicates.

Each dot represents the variance in expression for an individual mouse or *Anolis* gene. Genes are ordered based on their genome coordinates. To calculate variance, the count matrix containing raw counts was converted into a DESeqDataSet object. Then, the vst function in DESeq2 was used to normalize counts and stabilize variances before computing the variance using the apply function in R.

Table S1. Upregulated Hh responsive genes in ASEC-1 cell line after 400 nM SAG exposure for 48hr.

Available for download at
https://journals.biologists.com/dev/article-lookup/doi/10.1242/dev.204275#supplementary-data

Table S2. Number of *ift88* mutant clones and types of indels

No.	Clone ID	Type of indel	Biallelic and out-of-frame mutation
1	Clone #01	+ 1 / - 5	Yes
2	Clone #05	+ 1 / - 9	No
3	Clone #09	+ 1 / - 2	Yes
4	Clone #11	+ 1 / - 9	No
5	Clone #14	+ 227 / (WT/-1?)	No
6	Clone #15	+ 1 / - 9	No
7	Clone #19	+ 1 / - 9	No
8	Clone #25	- 1 / - 7	Yes
9	Clone #28	- 2 / - 4	Yes
10	Clone #29	+ 1 / - 9	No
11	Clone #30	+ 1 / - 5	Yes
12	Clone #31	+ 1 / - 1	Yes
13	Clone #32	+ 1 / - 9	No
14	Clone #33	+ 1 / - 1	Yes
15	Clone #41	+ 1 / - 1	Yes
16	Clone #42	+ 1 / - 4	Yes
17	Clone #43	+ 1 / - 5	Yes
18	Clone #44	+ 1 / - 1	Yes
19	Clone #45	+ 1 / - 5	Yes

Development • Supplementary information

Table S3. Digit numbers in polydactylous embryos from the eggs treated with 100 μM SAG

No.	Number of digits on hindlimbs		Number of digits of forelimbs		Developmental stage
	Left	Right	Left	Right	
1	5	5	5	5	Late 16
2	6	6	5	5	13
3	6	6	6	6	Late 13
4	6	6	6*	5*	14
5	6	6	6	6	13
6	6	6	5	5	14
7	6	6	5	5	13
8	6*	9*	6	6	Late 11
9	6	6	5	5	Late 13

* Asymmetry observed between left and right digit numbers

Table S4. Differentially expressed genes in response to 200 nM SAG exposure for 24 hr in *A. sagrei* and *M. musculus* primary limb cells.

Available for download at
https://journals.biologists.com/dev/article-lookup/doi/10.1242/dev.204275#supplementary-data

Development • Supplementary information

Table S5. Validation of RNAseq data by qRT-PCR

**Embryo no.**	**Fold induction relative to DMSO control**			
	gli1	*ptch1*	*cldn1*	*ramp2*
1	12.69	2.81	1.37	1.64
2	65.79	9.94	2.16	3.81
3	66.02	9.56	1.25	3.52
***p*-value =**	0.00014	0.00408	0.01709	0.00729

p values are associated with a two-tailed paired *t*-test. The analysis was performed using delta ct values.

Table S6. List of primer sequences used

Gene name	Primer name	Sequence
qRT-PCR primers		
gapdh	A.car-gapdh-fwd	ATCGGAGTCAACGGATTTGG
	A.car-gapdh-rev	CATGTAGACCATGTAGTTCAGG
tbp	A.sag-tbp-fwd	TCTCCAATGACTCCCATGAC
	A.sag-tbp-rev	CAGCCAAGATTTACCGTAGA
atpf1d	A.sag-atpf1d-fwd	AGACTCTTCCGTCCAACTCC
	A.sag-atpf1d-rev	TCAGACAAGGCCTTCTCCAG
gli1	A.sag-gli1-fwd	GCTCAGTACATGCTGGTTGTC
	A.sag-gli1-rev	CCGTGAGTAGGCTTTATTGCAG
ptch1	A.sag-ptch1-fwd	GTGGAGTTTACGGTTCACATTG
	A.sag-ptch1-rev	CACAGGTGCAAACATGTGTTCT
ramp2	A.sag-ramp2-fwd	AGTGGCAGGGTGAGCAAAG
	A.sag-ramp2-rev	GGTCCTCCTTCCAAAGACAA
cldn1	A.sag-cldn1-fwd	TGAAGTCAAGAAGATGAGGATG
	A.sag-cldn1-rev	AGTGAATGGGTTGAAGAACTC
Genotyping and sequencing primers		
ift88 (PAGE primers)	ift88-screen-F1	GATAATTCTGAATTAATTATAAACTCTTG
	ift88-screen-R1	TAACATTTAGGGCTCACTGG
ift88 (Sanger sequencing)	ift88-screen-F2	GTTATCAGTAGGAGGCAGTC
	ift88-screen-R3	CTCACTGGCTGAAATGCTGAG
ift88 (cDNA Sanger sequencing)	*Asag_ift88_Ex1-F1*	CTGAAGCAGATGAAGATGATC
	Asag_ift88_Ex8-R3	TCCAGCATCTTTTGCCTTCT
ift88 (Illumina sequencing)	*AS_ift88_illuminaseq_F2*	ACACTCTTTCCCTACACGACGCTCTTCCGATCTGT TATCAGTAGGAGGCAGTC
	AS_ift88_illuminaseq_R3	GACTGGAGTTCAGACGTGTGCTCTTCCGATCTCT CACTGGCTGAAATGCTGAG
Sex-genotyping primers		
AsagB-F1		GAAGACCAGGAGAGCAARGTC
AsagB-R1		GATGTCGGCAGCYTTGCGTAC
kank1-AcF		CCTTCCTTTGTAGGATCCAGTG
kank1-AcR		GGAGCACAGGGATAGTTTTGAC

Development • Supplementary information
