# Supplementary material for: A new cell culture resource for investigations of reptilian gene function
Source: Development. 2024 Nov 22;151(22):dev204275. doi: 10.1242/dev.204275 (PMC11607698; doi:10.1242/dev.204275)
Supplement: Supplementary file 1 [file develop-151-204275_review_history.pdf]

A new cell culture resource for investigations of reptilian gene function

Sukhada P. Samudra, Sungdae Park, Elizabeth A. Esser, Tryggvi P. McDonald, Arianna M. Borges, Jonathan Eggenschwiler and Douglas B. Menke

DOI: 10.1242/dev.204275

Editor: Cassandra Extavour

Review timeline

Original submission:	2 October 2023
Editorial decision:	27 November 2023
Resubmission:	25 July 2024
Accepted:	20 October 2024

Original submission

First decision letter

MS ID#: DEVELOP/2023/202385

MS TITLE:A new cell culture resource for investigations of reptilian gene function

AUTHORS: Sukhada P. Samudra, Sungdae Park, Elizabeth A. Esser, Tryggvi P. McDonald, Arianna M. Borges, Jonathan Eggenschwiler and Douglas B. Menke

Dear Dr. Menke,

I have now received all the referees' reports on the above manuscript, and have reached a decision. The referees' comments are appended below, or you can access them online: please go to BenchPress and click on the 'Manuscripts with Decisions' queue in the Author Area.

As you will see, the referees express considerable interest in your work, but have some significant criticisms and recommend a substantial revision of your manuscript before we can consider publication. The most important point is to significantly expand on the level of technical detail included about the development, maintenance and manipulation of the cell lines, as the generation of this new resource is the element of primary interest for this MS.

Previous Reviews

Reviewer 1 Advance Summary and Potential Significance to Field:

In the manuscript titled “A new cell culture 1 resource for investigations of reptilian gene function” Samudra et al., describe the development of reptilian in ovo and in vitro research tools/protocols. Specifically, they have investigated the in ovo response to stimulation of the Hedgehog pathway in Anolis sagrei, the brown anole lizard. Treating A. sagrei embroys with Smoothened agonist (SAG) leads to polydactyly associated with upregulation of Hedgehog transcriptional targets. The

© 2024. Published by The Company of Biologists under the terms of the Creative Commons Attribution License (https://creativecommons.org/licenses/by/4.0/).

transcriptomic responses to SAG between A. sagrei and M. musculus embryos are of similar developmental stages. The transcriptomic analyses from limb tissue revealed that amongst the transcripts commonly regulated by SAG in both A. sagrei and M. musculus, 84% are regulated in the same direction, either up- or down-regulated. This experiment demonstrated the common gene regulation network downstream of Hedgehog. The authors also successfully generated a clonal cell line (ASEC-1) cell line from primary fibroblasts, characterized its response to SAG, mapped the genome, created an ift88 mutant using CRISPR/Cas9 (also demonstrates that the cells can be used for transfections) and determined that primary cilia are required for Hh signaling in these cells. Overall, the authors have created an important tool for the research community. Given that this manuscript was submitted to be considered as a "Techniques and Resource" the paper would further benefit if more details and analyses outlined below are provided.

Reviewer 1 Comments for the Author:

Major concerns

• The manuscript would benefit with a more detailed description of the ASEC-1 cell line. We recommend DIC images, cell count at confluency, doubling time, time to passage, ratio to passage at and the recovery of the cell line when thawed.

• Line 576: There is very minimal information of how the cells were handled until passage 6. A reference is provided, however given that this is from a different animal, detailed description is warranted. What ratio were they split at? How were the primary LEFs treated to separate the tissue structures? Was trypsin used or any mechanical force used to disrupt the tissue? How many trials were undertaken and how many were successful? Was each trial a single embryo or multiple embryos (if so, how many)?

• Lines 200 and 262: How many single cells were plated? What is the success rate of generating single cell clones?

• Comparing the transcriptional profile of the primary LEF's (Fig2A) and ASEC-1 -/+ SAG will be a very instructional comparison. This analysis will provide insights into if and how the transcriptome changes upon immortalization, which is not a trivial question. In addition, the similarity of the response to SAG will be informative, increasing the utility of ASEC-1.

• Line 213: Which passage of ASEC-1 was used for transcriptomic analysis?

• Table S4 and Figure 5: No rationale is provided for the use of 400 nM of SAG or the amount used in any of the experiments.

• Lines 532, 582, 585: It would be important to report the number of days from the beginning of the experiment, i.e., Day 1 being when the primary LEF's are harvested.

• Line 163: There is no data provided supporting the statement that gene expression varied to a greater extent in lizard biological samples. It would also be appropriate NOT to use "our" to describe biological samples.

• Lines 263-264: The text here describes 19 single cell clones with 17 of them carrying biallelic frame-shifting mutations. However, Table S5 lists only 12 clones with biallelic frameshift mutations. It would be useful to identify (clone #) which clones have which mutations in table 5 rather than the first column that is only a numerical list. For instance, which of the clones listed in table 5 are #12 and #35? Since these are both wildtype, it is not clear if they are a part of this list or not.

• It is unclear from Figure 2A, B, results, methods, or statistics what the threshold is for considering a transcript as differentially regulated.

• There are several discrepancies with referencing

© 2024. Published by The Company of Biologists under the terms of the Creative Commons Attribution License (https://creativecommons.org/licenses/by/4.0/).

- Lines 77 and 440: Lewis et al., 1999 do not describe lack of cilia in Talpid3 mutants. A second paper from Yin et al., 2009 from the same group describe the loss of cilia phenotype
- Lines 981 and 984: Lozito et al., references incomplete
- Line 433: Sentence starting with “Mutant mouse…” – list reference
- Line 446: Sentence starting with “The derepression…” – list reference
- Line 434: The sentence describes interplay between Gli2 and Gli1 upon loss of cilia. However, some of the references refer to interplay between Gli3 and Gli1 (Haycraft et al., and Jia et al.,)
- In summary, would be important to go through the references and ascertain that the correct references are being used.

Minor concerns

• Error bars missing Fig 1D, ptch1 expression

• Line 542: Details of which genes were assessed from the extracted RNA and how would help.

• Lines 110, 205: The full form of the abbreviations need to be listed

• Line 440: “Such mutants” – list exactly which mutants are being referred to here.

• The discussion needs to comment more about the transcriptional profile of ASEC-1 and what the cell line can be characterized as in comparison to similar cell lines derived from mouse fibroblasts.

Reviewer 2 Advance Summary and Potential Significance to Field:

Samudra et al. describe a continuous cell line, called ASEC-1, that was derived from lizard (Anoles sagrei) embryonic fibroblasts (LEFs). The cells show morphological characteristics and gene expression patterns expected of fibroblasts. The authors use the cells and mutant derivatives lacking cilia (ift88 mutants) to establish that primary cilia are required for Hedgehog (Hh) signaling.

To provide background for the work, the team first establish some parameters of Hedgehog (Hh) signaling in lizards. These include showing that polydactyly is induced by the Smoothened agonist (SAG) and that transcriptional changes in SAG- treated primary LEFs are similar to those occurring in mouse embryonic fibroblasts (MEFs). The work allows a comparative analysis of vertebrate Hh signaling. The new cell line is a significant resource for the field and has already been used to study evolution of myoblast fusion in vertebrates (the publication is referenced in the manuscript—Zhang et al. 2022, DOI: 10.1126/sciadv.add2696). Having a squamate cell line should enable additional studies for which an in vitro model is useful.

Reviewer 2 Comments for the Author:

Overall, the paper is well written and therefore easy to read. The figures are excellent and so clear that they almost do not need legends. However, the organization of the manuscript does not reflect a paper describing a new resource. The title and abstract are focused on the resource—the new cell line, whereas the introduction is centered on Hh signaling and the brown anole as a new vertebrate model amenable to CRISPR and with a reference genome. This does establish the significance of the lizard for studying vertebrate Hh signaling but makes the cell line appear secondary. Editing to reverse this order—would be in keeping with this being a resource paper describing the ASEC-1 cells. It would also likely require a more lengthy discussion in general terms about the value of in vitro models for analyzing signaling. Studying Hh signaling in the cells can then be presented as an example of the biological value of the cells; however, some details about Hh signaling could be deferred to the discussion, or a combined results and discussion section. In the current version these sections are in some instances redundant.

© 2024. Published by The Company of Biologists under the terms of the Creative Commons Attribution License (https://creativecommons.org/licenses/by/4.0/).

Similarly, the order of the results does not fit with this being a paper about a resource. While I appreciate that the authors are laying some groundwork for studying Hh signaling in the cells (with the embryo and LEF experiments), the generation of the cells and their characteristics should be first. These data are straightforward, comprehensive (for example, includes genomic sequencing), and well described.

The data on treatment of eggs with SAG to induce polydactyly are also convincing; however, this could be a supplementary figure establishing the use of SAG for inducing the Hh pathway in the cells. The LEF analysis is important as it looks at global Hh signaling in cells. The logic for why this is done in primary cells should be clearer—are these cells rather than ASEC-1 cells used because they are not immortal and more like in vivo counterparts? I also wondered about the number of gene expression changes, which is much lower in the lizard—does this reflect overall gene number in the two species?

The results on Hh signaling and primary cilia are an excellent test case for the use of the cells in cell-biological research. The images and molecular data are convincing. Showing the cells are amenable to CRISPR will also be of interest to others wanting to use the cells.

If the Results and Discussion are combined, the authors could still end on a discussion point about the use of the cells to study Hh signaling and the conclusion about the lizard signaling in comparison with zebrafish, chick, and mouse. They could also highlight in more detail the interesting finding that cells in a derivative line differentiate into muscle and have been used to study the appearance of genes involved in myoblast fusion in vertebrate phylogeny.

Small point: qRT-PCR should be RT-qPCR.

Reviewer 3 Advance Summary and Potential Significance to Field:

This paper adapts the pharmacological activation of Hedgehog (Hh) signaling, originally demonstrated in mice, to Anolis sagrei (brown anole lizard), utilizing Smoothened Agonist (SAG). The emergence of a polydactyly phenotype in response to SAG exposure establishes Anolis sagrei as a promising model for investigating Hh signaling in reptiles. A comprehensive transcriptional analysis comparing responses in Anolis and mice limb cells unveils both conserved and unique gene expression patterns. The establishment of the ASEC-1 cell line emerges as a valuable resource, facilitating biological studies in reptiles and contributing significantly to our understanding of HH signaling and development. This cell line not only enhances our insights into Hh signal transduction in lizards but also paves the way for future explorations in reptilian biology.

Reviewer 3 Comments for the Author:

Major revision:

In the transcriptional analysis of ASEC-1 WT and mutant cell lines in figure 5, the focus is currently on measuring upregulated genes. However, it is crucial to note that the paper mentions the downregulation of Hip1 in LEFs following SAG treatment. To enhance the comprehensiveness of the

© 2024. Published by The Company of Biologists under the terms of the Creative Commons Attribution License (https://creativecommons.org/licenses/by/4.0/).

findings and strengthen the value of the ASEC-1 cell lines, it is recommended to include data on downregulated genes, particularly Hip1, in Figure 5 or any other relevant section. This additional information would not only provide a more holistic view of gene expression changes but also contribute to a deeper understanding of the role of ift88 in Hedgehog signaling, especially considering potential commonalities in downregulated genes between LEFs and MEFs

Minor revisions:

This paper introduces a valuable new cell line to the scientific community. To enhance the utility of this resource for future studies, it is suggested to include more technical details, such as:

• Providing information on the doubling time of the ASEC-1 line.

• Specifying the growing medium used for cell culture.

• Clarifying if cells are consistently grown at 29 degrees or under what conditions.

• Including details on transfection efficiency and whether other transfection methods were tested.

• Elaborating whether the CAG promoter was the sole pol II promoter tested, and if others were tested, providing a comparison of their strengths.

• Describing the process for freezing and thawing cells.

• Indicating the pol III sequence for guide RNA expression.

• Mentioning if other antibiotic selection reagents were optimized and, if so, providing relevant details.

Inclusion of these technical specifics would significantly benefit the scientific community, ensuring a more comprehensive understanding of the ASEC-1 cell line and maximizing its value as a resource for future research.

Author response to reviewers' comments

Response to Reviewers

We thank the reviewers for their comments and feel that the revised manuscript is substantially improved over our initial manuscript.

© 2024. Published by The Company of Biologists under the terms of the Creative Commons Attribution License (https://creativecommons.org/licenses/by/4.0/).

Reviewer 1 Comments for the Author:
Major concerns

•The manuscript would benefit with a more detailed description of the ASEC-1 cell line. We recommend DIC images, cell count at confluency, doubling time, time to passage, ratio to passage at and the recovery of the cell line when thawed.

As requested, additional characterization of ASEC-1 cell line is now documented in supplementary figure S2. This information is now mentioned on lines 94-96:

“ASEC-1 was characterized with respect to cellular morphology, doubling time, density at confluence, survival after freezing, sensitivity to puromycin, and efficiency of transfection using cationic lipids (Fig. S2).”

•Line 576: There is very minimal information of how the cells were handled until passage 6. A reference is provided, however given that this is from a different animal, detailed description is warranted. What ratio were they split at? How were the primary LEFs treated to separate the tissue structures? Was trypsin used or any mechanical force used to disrupt the tissue?

In the revised manuscript, we provide a more detailed technical description that addresses the reviewer’s questions. This information can be found in the method section on lines 519-556 under the heading “**Culturing of primary lizard fibroblasts**” and on lines 619-652 under the heading “**Generation of immortalized lizard embryonic fibroblast cell lines**.”

How many trials were undertaken and how many were successful? Was each trial a single embryo or multiple embryos (if so, how many)?

We started with a single embryo (one trial) and focused on tissue from the torso region. This trial was successful in generating immortalized cell lines. We mention that the ASEC-1 cell line was generated from a single embryo in text (Line 620-622). We believe the more detailed description of the immortalization procedure that we have included in the revised manuscript helps to clarify this (see above).

•Lines 200 and 262: How many single cells were plated? What is the success rate of generating single cell clones?

Information concerning the success rate of isolating immortalized clones is provided on lines 78- 79: *“192 single cells were sorted into 96-well plates. This ultimately resulted in the generation of approximately 40 surviving cell lines from the Anolis embryo.”*

Data addressing the successful isolation of clones after Cas9 transfection is provided in text on line 147-150:

“After transfecting ASEC-1 cells with Cas9 and ift88 gRNA expression constructs, we sorted 192 individual cells into 96-well plates, which resulted in the successful isolation and propagation of 46 clonal cell lines.”

•Comparing the transcriptional profile of the primary LEF’s (Fig2A) and ASEC-1 -/+ SAG will be a very instructional comparison. This analysis will provide insights into if and how the transcriptome changes upon immortalization, which is not a trivial question. In addition, the similarity of the response to SAG will be informative, increasing the utility of ASEC-1.

Determining how the transcriptome changes upon immortalization is a fascinating question. However, our data cannot easily address this issue. The primary LEF RNA-seq data were collected from limb LEFs, while the immortalized ASEC-1 cell line was generated from torso LEFs. Given that different tissues were used in these two sets of experiments and given the initial cellular heterogeneity in the primary cultures, we can not distinguish gene expression differences related to the immortalization process from those related to cell or tissue-type differences. Therefore, we have not included a direct transcriptome comparison between the primary limb LEFs and the

© 2024. Published by The Company of Biologists under the terms of the Creative Commons Attribution License (https://creativecommons.org/licenses/by/4.0/).

torso-derived ASEC-1 cell line.

•Line 213: Which passage of ASEC-1 was used for transcriptomic analysis?

We used passage # 34 and have added this information on line 809.

•Table S4 and Figure 5: No rationale is provided for the use of 400 nM of SAG or the amount used in any of the experiments.

We have added the rationale behind the SAG concentration used to collect data for this Figure (which is now Figure 1 in the revised manuscript). We initially used 200 nM of SAG to examine Hh responsiveness in lizard cell lines since published work found that Mouse NIH3T3 cells show their strongest responses to SAG at 100-200 nM (PMID: 12391318). This same dose was used for RNA-seq experiments on primary limbs cells from lizard and mouse. For comparing Hh responsiveness in wild type and *ift88* mutant ASEC-1 cell lines, we increased the dose to 400 nM SAG because we determined that wild-type ASEC-1 cells have a stronger response at this dose. We have included the rationales for the SAG concentrations used in different experiments as follows:
Line 82-84: *"We subjected the cell lines to 200 nM SAG for 24 hours; these conditions are sufficient to induce Hh signaling in immortalized mouse fibroblasts (Chen et al., 2002)."*

Line 170-175: *"The SAG responsiveness of wild-type ASEC-1 cells, as assessed by gli1 and ptch1 induction, was robust at 400 nM SAG concentration and 24 hours of SAG exposure (Fig. 1B,C; Fig. S1). Hence, we chose to compare the SAG response of the parental cell line ASEC- 1, clone #12 ift88$^{+/+}$, clone #35 ift88$^{+/+}$, clone #28 ift88$^{-2/-4}$, and clone #30 ift88$^{+1/-5}$ at 400 nM SAG for 24 hours ."*

The rationale behind the SAG concentration used in for in ovo Induction of Hh signaling is provided on Lines 502-504: *"Preliminary experiments indicated that treatment of eggs with a single bolus of 100 μM SAG or higher was sufficient to consistently induce changes in digit number."*

•Lines 532, 582, 585: It would be important to report the number of days from the beginning of the experiment, i.e., Day 1 being when the primary LEF's are harvested.

We added details in each method section that include the time it took to passage the cells and generate data for primary LEFs and MEFs and the ASEC-1 cell line. The details are below (see manuscript for complete passages):

Line 558-566:
Pharmacological manipulation of Hh signaling pathway in primary LEFs
"Primary limb and torso LEFs were collected from individual A. sagrei stage 6 embryos. Primary LEFs from an individual embryo were grown until passage 3 (primary limb LEFs ~ 10-12 days; primary torso LEFs ~ 7-8 days, counted from the initial collection of the primary cells from the embryo)...Primary limb LEFs required ~3-4 days and primary torso LEFs required ~2-3 days to become at least 70% confluent at each passage."

Line 578-586:
RNA-seq analysis of SAG responsive genes in *A. sagrei* and *M. musculus*
"Primary limb LEFs were disaggregated as described above. We plated passage 0 cells in a 24-well plate. After 4-5 days, when the cells became confluent, cells were detached using trypsin and replated in a 6-well plate as described in methods for culturing primary LEFs. This was passage 1. For passage 4, the cells were split into two wells in a 6-well plate. Primary limb LEFs required ~8 days to become at least 70% confluent between the passages…Primary limb MEFs required 2-3 days to become at least 70% confluent between the passages."

Line 618-652:
Generation of immortalized lizard embryonic fibroblast cell lines
"Passage 0 cells were plated in a 24-well plate. When the cells reached ~80% confluency after 3-4 days, they were trypsinized and plated again in a 12-well plate (described in detail in the method for culturing of primary cells). This was passage 1… Within 3-4 days the cells became

© 2024. Published by The Company of Biologists under the terms of the Creative Commons Attribution License (https://creativecommons.org/licenses/by/4.0/).

confluent and were transitioned to a 6-well plate. This was considered passage 2…At passage 26 very few cells were attached to the plate, and there were no obvious signs of cell growth for two weeks. This process took ~4.5 months from the day we harvested primary LEFs…After this "no growth period", visible cell growth was observed in the form of patches or colonies of cells. The few cells which escaped cellular senescence started growing at this point. We allowed these cells to grow and become confluent. Within 7-8 days of first observing patches of cell growth, the cells in the 10 cm plate were confluent. Cells were then trypsinized, and single cells were sorted…The complete process of generating and expanding single-cell clones starting from the day we harvested the primary cells took ~6 months."

•Line 163: There is no data provided supporting the statement that gene expression varied to a greater extent in lizard biological samples. It would also be appropriate NOT to use "our" to describe biological samples.

In our initial submission, we performed a statistical test (Bartlett's test of equal variance) that assesses whether two datasets exhibit the same level of variation (line 250 of the revised manuscript). The analysis provided strong statistical support ($p < 2.2 \times 10^{-16}$) for our conclusion that variance is elevated in the lizard samples relative to the mouse samples. The underlying data are the RNA sequence counts for all genes from every library (hundreds of thousands of data points), and the statistical test does not produce a graphical output to display to the reviewer. However, to display the variance in the data we have now also included a variance plot that includes variation for every expressed gene in the mouse and *Anolis* RNA-seq datasets. This plot visually demonstrates that the level of variation in the *Anolis* RNA-seq replicates is much higher than that observed for the mouse RNA-seq replicates (Figure S3). We also have removed the use of "our" when describing biological samples.

•Lines 263-264: The text here describes 19 single cell clones with 17 of them carrying biallelic frame-shifting mutations. However, Table S5 lists only 12 clones with biallelic frameshift mutations. It would be useful to identify (clone #) which clones have which mutations in table 5 rather than the first column that is only a numerical list. For instance, which of the clones listed in table 5 are #12 and #35? Since these are both wildtype, it is not clear if they are a part of this list or not.

We have reorganized the supplemental table (Table S2 in the revised manuscript) and now list the mutant clones individually rather than grouping them based on the type of mutations that they carry. We have also corrected the text to indicate that of the 19 mutant clones, 12 carry biallelic frameshift mutations.

Lines 150-152: *"PCR amplification of exon 4 followed by PAGE revealed 19 clonal cell lines with indels in ift88. Sanger sequencing demonstrated that 12 of these clones carried frame-shifting mutations on both ift88 alleles (Table S2)."*

•It is unclear from Figure 2A, B, results, methods, or statistics what the threshold is for considering a transcript as differentially regulated.

Note that in the revised manuscript, this figure is now Figure 5. In this figure we display all genes that have a statistically different level of expression between -SAG and +SAG conditions. In principle, even modest differences in expression might be biologically relevant. Therefore, rather than use an arbitrary fold difference threshold, we focus our analyses in Figure 5C and 5D on genes that display a statistically significant SAG response in both lizard and mouse cells. Thus, we filter the DE gene list based on observation of a shared response between the two species. We have made the following changes to the text to make this clear:

Lines 260-262: "Rather than using an arbitrary fold-difference threshold to determine which genes are likely to be biologically relevant Hh-responsive genes, we focused on genes with are SAG responsive in both anole and mouse."

•There are several discrepancies with referencing

- **§ Lines 77 and 440: Lewis et al., 1999 do not describe lack of cilia in Talpid3 mutants. A second paper from Yin et al., 2009 from the same group describe the loss of cilia phenotype**

© 2024. Published by The Company of Biologists under the terms of the Creative Commons Attribution License (https://creativecommons.org/licenses/by/4.0/).

We revised and added the correct the reference provided on the line 382.

§ Lines 981 and 984: Lozito et al., references incomplete

During revisions, we removed this section to streamline the discussion and no longer cite this work.

§ Line 433: Sentence starting with "Mutant mouse…" - list reference

We have cited appropriate references for this statement (Line 375).

§ Line 446: Sentence starting with "The derepression…" - list reference

We have cited appropriate references for this statement (Line 388).

§ Line 434: The sentence describes interplay between Gli2 and Gli1 upon loss of cilia. However, some of the references refer to interplay between Gli3 and Gli1 (Haycraft et al., and Jia et al.,)

We have removed these references.

§ In summary, would be important to go through the references and ascertain that the correct references are being used.

We double checked our citations to ensure correct references are used.

Minor concerns
•Error bars missing Fig 1D, ptch1 expression

After revising the manuscript, this is now Figure 4D. We used R to produce graphical representations of our data. In Figure 4D, we have five data points for *ptch1* (n= 5). Out of these 5 data points, two are outliers as shown in the figure. In the *ptch1* box plot, the minimum value data point happens to be equal to the lower quartile and the maximum value data point is equal to the upper quartile, thus the whiskers have zero length and, hence, are not plotted.

•Line 542: Details of which genes were assessed from the extracted RNA and how would help.

We now list these details under the subheading "**RNA isolation and qRT-PCR**" (Lines 769- 792). We also refer to this section in the text at the end of the section titled "**Pharmacological manipulation of Hh signaling pathway in primary LEFs**" (Line 576).

•Lines 110, 205: The full form of the abbreviations need to be listed

We have added the full name of the ASEC-1 cell line (Line 87) and Hh (Hedgehog; Line 60)

•Line 440: "Such mutants" - list exactly which mutants are being referred to here.

We were referring to chicken *Talpid3* mutants. We have updated the text to make this clear:

Lines 379-382: *"Chicken Talpid3 mutants lacking cilia show dramatic loss of expression of Hh target genes in the neural tube…"*

•The discussion needs to comment more about the transcriptional profile of ASEC-1 and what the cell line can be characterized as in comparison to similar cell lines derived from mouse fibroblasts.

Direct comparisons of ASEC-1 global transcriptome data to that of different mouse fibroblast lines is challenging due to the large evolutionary distance between these species. However, we have added a paragraph to the discussion that is focused on the expression of marker genes that can

© 2024. Published by The Company of Biologists under the terms of the Creative Commons Attribution License (https://creativecommons.org/licenses/by/4.0/).

be used to distinguish mammalian fibroblasts, mural cells, and myofibroblasts. We tentatively suggest that expression profile of ASEC-1 cells are most similar to mammalian myofibroblasts:

Lines 319-339: *"Based on their expression profile, we suggest that ASEC-1 cells are best described as myofibroblasts…but given the large evolutionary distance between lizards and mammals, we view this conclusion as tentative."*

****

Reviewer 2 Comments for the Author:
Overall, the paper is well written and therefore easy to read. The figures are excellent and so clear that they almost do not need legends. However, the organization of the manuscript does not reflect a paper describing a new resource. The title and abstract are focused on the resource—the new cell line, whereas the introduction is centered on Hh signaling and the brown anole as a new vertebrate model amenable to CRISPR and with a reference genome. This does establish the significance of the lizard for studying vertebrate Hh signaling but makes the cell line appear secondary. Editing to reverse this order—would be in keeping with this being a resource paper describing the ASEC-1 cells. It would also likely require a more lengthy discussion in general terms about the value of in vitro models for analyzing signaling. Studying Hh signaling in the cells can then be presented as an example of the biological value of the cells; however, some details about Hh signaling could be deferred to the discussion, or a combined results and discussion section. In the current version these sections are in some instances redundant.

In response to the reviewer's comments, we have shortened and refocused the introduction on the need for new cell culture resources for reptiles. We also, very briefly, mention the additional objective of generating new transcriptome resources for comparative studies of hedgehog signaling.

Similarly, the order of the results does not fit with this being a paper about a resource. While I appreciate that the authors are laying some groundwork for studying Hh signaling in the cells (with the embryo and LEF experiments), the generation of the cells and their characteristics should be first. These data are straightforward, comprehensive (for example, includes genomic sequencing), and well described.

As requested, we have reversed the order of the results. In the revised manuscript generation of the immortalized cell line is presented first.

The data on treatment of eggs with SAG to induce polydactyly are also convincing; however, this could be a supplementary figure establishing the use of SAG for inducing the Hh pathway in the cells. The LEF analysis is important as it looks at global Hh signaling in cells. The logic for why this is done in primary cells should be clearer—are these cells rather than ASEC-1 cells used because they are not immortal and more like in vivo counterparts? I also wondered about the number of gene expression changes, which is much lower in the lizard—does this reflect overall gene number in the two species?

We have added additional text throughout the manuscript to make the point that part of our objective is to generate resources for comparative studies of hedgehog signaling (e.g., see Lines 63-69). Most of our knowledge of hedgehog signaling comes from major model systems, including mouse. The use of primary lizard and mouse limb cells allowed us to generate parallel datasets from homologous cell populations from these two species and provides some initial insights into conserved Hh transcriptional responses between mammals and reptiles. For cross species comparisons of Hh responsive genes, immortalized cell lines are probably less ideal for a variety of reasons, including different tissue or cell type origins of the lines, different methods of immortalization, and dramatically different amounts of time spent in culture (passage # since the cell lines were created). We include an extensive discussion of these points in in our revised manuscript (Lines 399-469)

Concerning the lower number of gene expression differences that we detected in anoles as compared to mouse, this is not due to differences in gene number. The number of protein coding genes in anoles and mouse are very similar (~20,000 protein coding genes in each species). As we mention in the manuscript, there is much greater variation between the lizard RNA-seq replicates

© 2024. Published by The Company of Biologists under the terms of the Creative Commons Attribution License (https://creativecommons.org/licenses/by/4.0/).

than the mouse replicates; this high variability in the expression of individual genes means that we are less likely to detect significant expression differences in the lizard dataset, especially for genes that have a modest response to SAG. The source of this greater gene expression variation is likely a combination of much higher levels of genetic variation between individual lizard embryos and a greater spread in the developmental stages used.
Consistent with this hypothesis, we note that our ASEC-1 cell line RNA-seq replicates show minimal variation between replicates (there should be virtually no genetic variation between the ASEC-1 replicates). Thus, the differences in statistically different gene expression changes is not simply a result of the number of genes in the lizard and mouse genome, but must result from other sources of variation.

The results on Hh signaling and primary cilia are an excellent test case for the use of the cells in cell-biological research. The images and molecular data are convincing. Showing the cells are amenable to CRISPR will also be of interest to others wanting to use the cells.

If the Results and Discussion are combined, the authors could still end on a discussion point about the use of the cells to study Hh signaling and the conclusion about the lizard signaling in comparison with zebrafish, chick, and mouse. They could also highlight in more detail the interesting finding that cells in a derivative line differentiate into muscle and have been used to study the appearance of genes involved in myoblast fusion in vertebrate phylogeny.

Small point: qRT-PCR should be RT-qPCR.

Both abbreviations are commonly found in the scientific literature and appear to be used interchangeably. We used the article search function at the journal's website to determine which abbreviation is preferred at *Development*. "qRT-PCR" is more commonly used in *Development* articles so we have opted to use that abbreviation.

****
Reviewer 3 Comments for the Author:
Major revision:

In the transcriptional analysis of ASEC-1 WT and mutant cell lines in figure 5, the focus is currently on measuring upregulated genes. However, it is crucial to note that the paper mentions the downregulation of Hip1 in LEFs following SAG treatment. To enhance the comprehensiveness of the findings and strengthen the value of the ASEC-1 cell lines, it is recommended to include data on downregulated genes, particularly Hip1, in Figure 5 or any other relevant section. This additional information would not only provide a more holistic view of gene expression changes but also contribute to a deeper understanding of the role of ift88 in Hedgehog signaling, especially considering potential commonalities in downregulated genes between LEFs and MEFs

While *hip1* is downregulated in response to SAG in primary limb LEFs and MEFs, it does not respond to SAG in the parental ASEC-1 cell line. In general, genes that are down-regulated in response to SAG show much more modest fold changes (<2X) than those genes that are upregulated in response to SAG. Therefore, we have chosen not to assess down-regulated genes in the *ift88* mutant clones. We do, however, provide a complete list of genes that are down regulated in response to SAG in primary limb LEFs, MEFs, and ASEC-1 for future investigations of Hh transcriptional responses in anoles or mouse.

Minor revisions:

This paper introduces a valuable new cell line to the scientific community. To enhance the utility of this resource for future studies, it is suggested to include more technical details, such as:

•Providing information on the doubling time of the ASEC-1 line.

This information has been added to the manuscript and can be found in Figure S2.

© 2024. Published by The Company of Biologists under the terms of the Creative Commons Attribution License (https://creativecommons.org/licenses/by/4.0/).

•Specifying the growing medium used for cell culture.
In our initial submission, we provided growth medium information in the method section for culturing primary lizard fibroblasts and then later stated that the same cell culture conditions were used for the cell line. To provide further clarity we have added the recipe again in the method section under the heading "**Generation of immortalized lizard embryonic fibroblast cell lines**" (Lines 633-637).
•Clarifying if cells are consistently grown at 29 degrees or under what conditions.

In the original manuscript, the growth conditions are mentioned in the method section for culturing primary lizard fibroblasts. We now mention the growth conditions again in the method section under the heading of generation of immortalized lizard cell lines (Line 645).

•Including details on transfection efficiency and whether other transfection methods were tested.

For this study, we only used lipofection. The transfection efficiency information has been added to a new supplemental figure (Figure S2). In previous work, we demonstrated that nucleofection can also be used on primary *Anolis* cells (Park et. al, 2014).

Park et. al (2014) *J Exp Zool B Mol Dev Evol*. 322(3):156-65.

•Elaborating whether the CAG promoter was the sole pol II promoter tested, and if others were tested, providing a comparison of their strengths.

For this study, we used CAGG-based Cas9 expression constructs because they have been used successfully to perform gene editing in human and mouse fibroblast cell lines.

•Describing the process for freezing and thawing cells.

In the revised manuscript, we have added this information to the methods section (Lines 667- 682).

•Indicating the pol III sequence for guide RNA expression.

For gRNA expression, we followed the design used by Mali et al., 2013.
Therefore, we have simply included the appropriate reference (Mali et al., 2013), which includes these design details, including the pol III sequence (Line 744).

•Mentioning if other antibiotic selection reagents were optimized and, if so, providing relevant details.

We only tested puromycin. The puromycin survival curve for ASEC-1 cells is now included in Figure S2.

Inclusion of these technical specifics would significantly benefit the scientific community, ensuring a more comprehensive understanding of the ASEC-1 cell line and maximizing its value as a resource for future research.

We agree and have added the requested details.

Resubmission

First decision letter

MS ID#: DEVELOP/2024/204275

MS TITLE:A new cell culture resource for investigations of reptilian gene function

© 2024. Published by The Company of Biologists under the terms of the Creative Commons Attribution License (https://creativecommons.org/licenses/by/4.0/).

AUTHORS: Sukhada P. Samudra, Sungdae Park, Elizabeth A. Esser, Tryggvi P. McDonald, Arianna M. Borges, Jonathan Eggenschwiler and Douglas B. Menke

Dear Dr Menke,

I am happy to tell you that your manuscript has been accepted for publication in Development, pending our standard publication integrity checks.

Comments from the Reviewers:

Reviewer 1: SUMMARY OF THE ADVANCE MADE IN THIS PAPER AND ITS POTENTIAL SIGNIFICANCE TO THE FIELD

This manuscript introduces ASEC-1, a novel immortalized lizard fibroblast cell line from Anolis sagrei. This cell line provides a new resource for studying reptilian gene function and conserved cellular processes, as demonstrated with Hh signaling. The study also demonstrates the utility of CRISPR/Cas9 mutagenesis in disrupting the ift88 gene to explore the role of cilia structure in Hh signaling. The cross-species comparison of SAG-induced transcriptional responses between A sagrei and M musculus provides evolutionary insights into the conserved and divergent aspects of Hh signaling.

The significance of this work lies in its potential to enhance the functional genomic toolkit available for reptilian studies. The study provides foundational resources for the comparative evolutionary and developmental biology communities and establishes A sagrei as a promising model for further genetic research.

SUGGESTIONS TO AUTHORS

No requested revisions.

The manuscript has clearly undergone substantial revision, and many concerns raised by previous reviewers have been addressed. The manuscript is well-written, clear, and concise. The figures and supplementary data are thorough and informative. The experimental design and methods are robust, and the authors have convincingly demonstrated the utility of ASEC-1 for CRISPR/Cas9 gene editing, even with a modest transfection efficiency. If any, I would think the 9% transfection efficiency is low, but they were able to isolate multiple KO clones from that.

Reviewer 2: SUMMARY OF THE ADVANCE MADE IN THIS PAPER AND ITS POTENTIAL SIGNIFICANCE TO THE FIELD

This will be the first lizard cell line. Its utility in studying Hh signaling has been explored.

SUGGESTIONS TO AUTHORS

The paper has been significantly revised in response to the previous review. The main point I raised about the focus needing to be on the cell line as a resource rather than Hh signaling has been addressed—it now reads as a resource paper. There are also many new details about the cell line including its derivation and maintenance, which will be very useful to researchers in the lizard research field who may be using cell culture for the first time, as this is the first lizard cell line! One small point: could the fact that puro selection was used to enrich for cells that had undergone

© 2024. Published by The Company of Biologists under the terms of the Creative Commons Attribution License (https://creativecommons.org/licenses/by/4.0/).

CRISPR-mediated gene editing be mentioned in the Results section. Currently, it seems as if the whole population was the starting point for single-cell selection (it is in the Methods but should also be in the Results).

© 2024. Published by The Company of Biologists under the terms of the Creative Commons Attribution License (https://creativecommons.org/licenses/by/4.0/).
